# Three-dimensional scanless patterned illumination using time-multiplexed multiline temporal focusing for multicell manipulation with single-cell resolution

**DOI:** 10.1117/1.JBO.30.7.075003

**Published:** 2025-07-28

**Authors:** Kenta Inazawa, Mayumi Yamada, Takayuki Michikawa, Kana Namiki, Atsushi Miyawaki, Itaru Imayoshi, Katsumi Midorikawa, Keisuke Isobe

**Affiliations:** a RIKEN Center for Advanced Photonics, Attosecond Science Research Team, Saitama, Japan; b Kyoto University, Graduate School of Biostudies, Laboratory of Brain Development and Regeneration, Kyoto, Japan; c Hamamatsu Photonics K.K., Electron Tube Division, Iwata, Japan; d Kyoto University, Institute for Life and Medical Sciences, Laboratory of Deconstruction of Stem Cells, Kyoto, Japan; e RIKEN Center for Advanced Photonics, Biotechnological Optics Research Team, Saitama, Japan; f Kyoto University, Institute for Life and Medical Sciences, Laboratory of Optical Biomedical Science, Kyoto, Japan; g RIKEN Center for Brain Science, Laboratory for Cell Function Dynamics, Saitama, Japan; h Kyoto University, Institute for Life and Medical Sciences, Laboratory of Bioresponse Analysis, Kyoto, Japan; i Kyoto University, Graduate School of Biostudies, Laboratory of Spatiotemporal Optical Control, Kyoto, Japan

**Keywords:** patterned illumination, photostimulation, temporal focusing

## Abstract

**Significance:**

Three-dimensional (3D) two-photon patterned illumination using a combination of computer-generated holography (CGH) and wide-field temporal focusing (TF) has emerged as a highly effective approach for photostimulation. However, even though the axial full-width at half-maximum of a single-spot by TF is smaller than the single-cell size of 10  μm, the axial resolution of 3D multispot patterns produced by CGH with TF is lower than the single-cell resolution as a result of interference among multispots.

**Aim:**

We aim to achieve 3D two-photon patterned illumination with single-cell resolution by combining CGH with time-multiplexed multiline temporal focusing (TM-ML-TF), which is implemented by adding an echelle grating at a position conjugate to the focal plane of the TF-CGH system.

**Approach:**

We measure the 3D two-photon fluorescence distributions generated by the TF-CGH and TM-ML-TF-CGH systems.

**Results:**

In TM-ML-TF, the crosstalk artifacts between the target spots in two proximal planes with an axial distance of 20  μm were suppressed from 81% to 15% compared with those in TF. We successfully achieved the photoconversion of 3D target cells in spheroids with single-cell resolution.

**Conclusions:**

TM-ML-TF successfully suppresses the interference among multispots, enabling the TM-ML-TF-CGH system to provide precise 3D patterned illumination with single-cell resolution.

## 1 Introduction

Optogenetics^1^^–^^3^ is a powerful tool for elucidating the link between neuronal circuit activity and animal behavior. Efficiently accomplishing this elucidation requires precise control of the firing timing of multiple target cells arranged in three dimensions by photostimulation with single-cell resolution while monitoring neuronal circuit activity. The two-photon (2P) excitation method^4^^,^^5^ with spatially tight focusing at a high numerical aperture (NA) can localize the photostimulated region to a single-cell size in the axial direction. However, activation by spatially tight focusing requires lateral scanning of the laser over a single cell. The illumination spot can be expanded to single-cell size by computer-generated holography (CGH);^6^ however, as with low-NA focusing, the axial excitation region increases in proportion to the spot size.^7^ In addition, the quality of the excitation beam worsens because of speckle noise resulting from the interference effect of phase modulation. Thus, the trade-off between temporal and axial resolution has become a significant obstacle.

The wide-field temporal focusing (TF) technique^8^^,^^9^ can mitigate this trade-off between temporal and axial resolution. In TF, spectrally dispersed pulses are generated by a diffraction grating placed conjugate to the focal plane of an objective lens. The local spectral bandwidth in the out-of-focus region is narrower due to spectral dispersion, and all the spectral components are recombined only at the focal plane. Furthermore, the group delay dispersion converted from the quadratic phase introduced by the objective lens is compensated for in its propagation to the focal plane. Thus, pulse width becomes a function of the propagation distance, and the shortest pulse duration can be achieved at the focal plane. The 2P excitation probability is inversely proportional to the pulse duration. Therefore, the 2P excitation region can be confined near the focal plane even if the spot is laterally expanded to a single-cell size. TF can also reduce speckle noise caused by scattering in tissue.^10^

Recently, several groups have demonstrated scanless patterned illumination systems that combine CGH with liquid-crystal-on-silicon spatial light modulators (LCOS-SLMs) and wide-field TF for the simultaneous stimulation of multiple cells.^11^^–^^18^ In this method, LCOS-SLMs function as beam shapers^11^^,^^12^ or beam splitters.^13^^–^^15^ When an LCOS-SLM is used for beam shaping,^11^^,^^12^ two-dimensional (2D) pattern light is generated by the LCOS-SLM placed in the front Fourier plane of the diffraction grating for TF. The axial confinement of 2D patterned photostimulation can be realized by the TF effect. However, speckle noise appears in the patterns. This problem can be overcome using the generalized phase contrast method with wide-field TF.^18^ When an LCOS-SLM is used as a beam splitter,^13^^–^^17^ multiple TF spots of the same shape are generated in three dimensions by the LCOS-SLM placed in the back Fourier plane of the diffraction grating for TF. Thus, three-dimensional (3D) patterned photostimulation can be achieved. A multishaped 2D pattern and multifocal 3D pattern can be generated by placing two LCOS-SLMs in the front and back Fourier planes of the diffraction grating.^16^^,^^17^

Nevertheless, several problems make accurate photostimulation of multiple target cells in three dimensions difficult. First, the axial resolution is insufficient for 10  μm single-cell resolution in photostimulation. The axial resolution of wide-field TF is less than that of spatially tight focusing, which is equivalent to that of line TF because wide-field TF lacks spatial focusing characteristics along the direction perpendicular to the spectrally dispersed direction.^19^^,^^20^ Even if the axial resolution of the wide-field TF evaluated in terms of the full-width at half-maximum (FWHM) is 5  μm, the ${1/e}^{2}$ width is calculated to be ∼20  μm using the following depth response equation:^20^

$$R_{TF}(z)=\frac{1}{\sqrt{1+(z/z_{R}{)}^{2}}},$$ (1)

where $z_{R}$ is related to the FWHM ($z_{R}=\mathrm{FWHM}/2\sqrt{3}$). Thus, the crosstalk artifacts between the target spots in two proximal planes with an axial distance of 20  μm are large because of the interference among the axial multispots. In addition, accurately generating a pattern designed to keep multitarget cells close together in the direction orthogonal to the spectral dispersion is difficult because of the interference of TF spots close to each other in this direction.^13^ The line focusing on the LCOS-SLM by TF also causes thermal effects in the LCOS-SLM and degrades hologram quality. Alternate current (AC) drive is usually used in liquid crystal devices because direct current drive causes a decrease in the response time as a result of the accumulation of ions on the electrode surface in the cell; it also causes degradation of liquid crystals as a result of electrolysis. When the AC drive frequency is sufficiently low for the liquid crystal molecules to follow, the phase modulated by the LCOS-SLM fluctuates periodically.^21^ This temporal fluctuation is known to increase with increasing heat of the LCOS-SLM.^22^ Because the local temperature increase depends on the illumination area, the line focusing on the LCOS-SLM causes a large temporal fluctuation. To avoid the line focusing on the LCOS-SLM, researchers have attempted to expand the beam spot size on the LCOS-SLM; however, another problem has arisen. When a spherical lens is introduced in front of the diffraction grating to expand the beam spot on the LCOS-SLM, the axial resolution is degraded because the focal plane of TF is not coincident with the spatial focal plane of the spherical lens.^14^ If the spatial coherence is reduced using a rotating diffuser to expand the beam spot on the LCOS-SLM,^15^ single-shot photostimulation is no longer possible because of the need for integration to remove speckle noise. In addition, the diffuser increases the overlap area of each spectral spot when out of focus, which reduces the axial confinement.

**Fig. 1:**
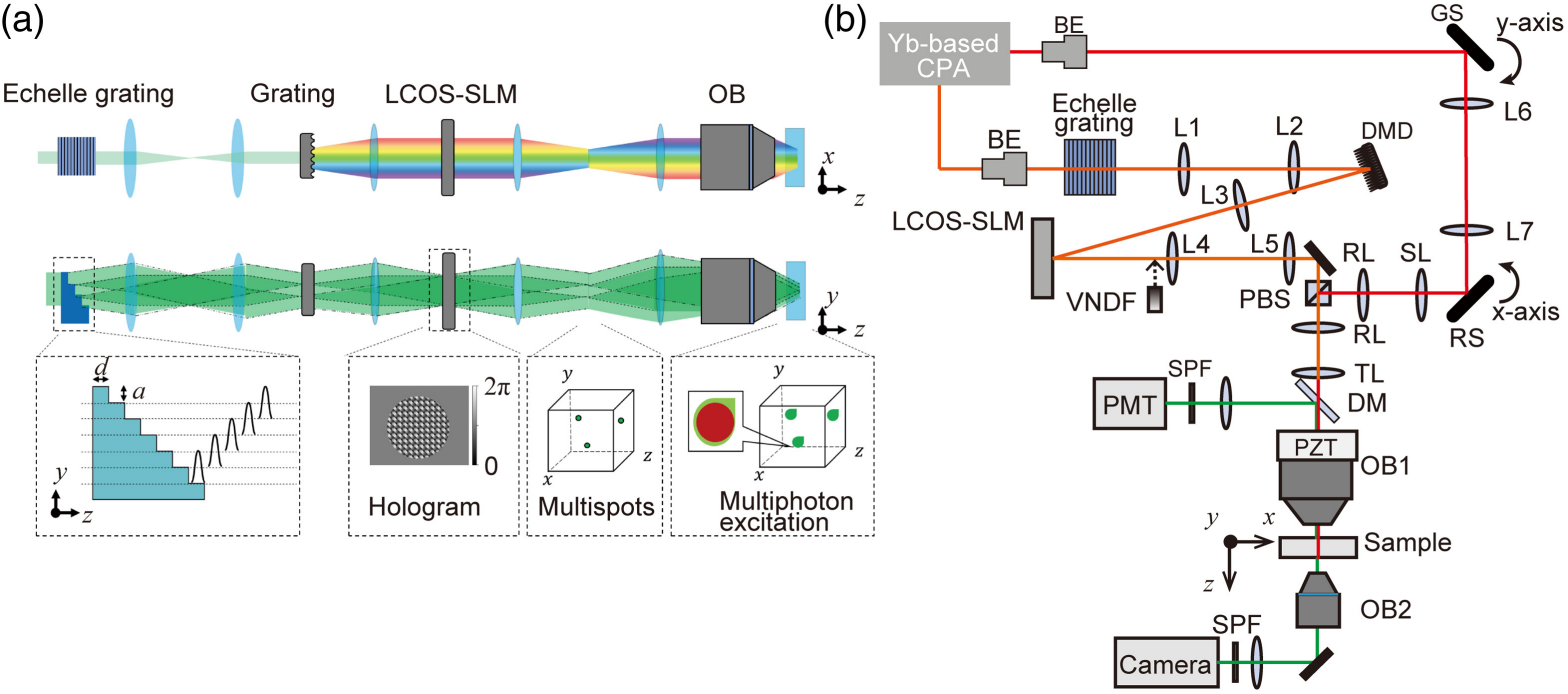
(a) Schematics of 3D patterned illumination with TM-ML-TF. An echelle grating produces multiple beamlets with a temporal delay among them, which is induced by the difference in the refractive index between the glass and air. Multiple beamlets are focused into a sample through three 4-f systems. A computer-generated hologram is displayed on an LCOS-SLM placed at the back Fourier plane of a diffraction grating to generate a multispot at the desired 3D location. The multitarget cells are photostimulated by multiphoton excitation with patterned light generated by the CGH phase mask. (b) Experimental setup for 3D patterned illumination with TM-ML-TF (orange line) and 2P laser scanning microscopy (red line). BE, beam expander; DMD, digital micromirror device; L, lens; LCOS-SLM, liquid-crystal-on-silicon spatial light modulator; VNDF, variable neutral-density filter; OB, objective lens; PZT, piezoelectric transducer; DM, dichroic mirror; SPF, short-pass filter; GS, galvanometric scanner; RS, resonant scanner; SL, scan lens; TL, tube lens; RL, relay lens; PMT, photomultiplier tube.

A photostimulation system involving the combination of time-multiplexed multiline temporal focusing (TM-ML-TF)^23^^–^^26^ and the CGH technique has the potential to eliminate the aforementioned issues. TM-ML-TF can be realized by placing an echelle grating conjugate to the focal plane of TF [Fig. 1(a)]. After a pulse propagates through each step of the echelle grating, the pulse splits into multiple pulses with a temporal delay among them. If the temporal delay is longer than the coherence time of the pulses, the neighboring pulses from each step of the echelle grating do not interfere with each other near the echelle grating. As a result, the spatial coherence decreases only in the direction along the steps of the echelle grating. Thus, the TM pulses from each step are diffracted and line-focused at each conjugate position of the focal plane of an objective lens through 4f optical systems. Because the echelle grating introduces spatial focusing properties along the direction perpendicular to the spectrally dispersed direction, the TM-ML-TF technique provides axial resolution comparable with that of line TF.^23^ The improved axial resolution with TM-ML-TF has been demonstrated in wide-field 2P TF imaging.^23^^–^^25^ However, the field of view (FOV) of TM-ML-TF microscopy has been limited to 6  μm in diameter^23^ and 20×15  μm.^24^ In our previous work,^26^ the FOV of TM-ML-TF microscopy was expanded to 70  μm in diameter by 2D patterned illumination combining TM-ML-TF with the CGH technique, in which eight spots arranged laterally around the center spot on a concentric circle were generated. The illumination area was expanded without causing fringes or speckles because interference among multispots in close proximity can be suppressed by temporal delays.^26^ Because the previous setup was dedicated to widening the FOV of TM-ML-TF microscopy, the aliasing-free lateral displacement of target spots by CGH was limited to ±79.5  μm, and the single illumination spot diameter was enlarged to 30  μm by relaxing the imaging magnification from the sample to the echelle grating. However, optical manipulation, including photostimulation, requires larger lateral and axial displacements without aliasing for a large field of excitation (FOE) and a single spot diameter of 10  μm with an axial confinement of 10  μm for 3D single-cell resolution. In addition, TM-ML-TF-CGH has not been applied to 3D patterned illumination, and the FOE in the axial direction and the crosstalk artifacts between two target spots in axially separated planes have not been evaluated. Although the beam size on the LCOS-SLM can also be increased by TM-ML-TF, the thermal effect of the LCOS-SLM has not been investigated.

Here, we demonstrate a photostimulation system that combines TM-ML-TF with the CGH technique to eliminate the aforementioned issues of TF-CGH with 3D patterned illumination. In this system, by optimizing the key parameters for 3D patterned illumination using TM-ML-TF-CGH, the aliasing-free lateral and axial displacements are extended to ±226 and ±155  μm, respectively, and the single spot diameter is set at 10  μm. We characterize the accessible FOEs in the lateral and axial directions. We demonstrate that TM-ML-TF-CGH has better resistance to the LCOS-SLM thermal effect, fewer crosstalk artifacts between two target spots in axially separated planes, and less degradation of axial resolution at large axial displacements compared with TF-CGH. We also show the photoconversion of 3D target cells in spheroids with single-cell resolution.

## 2 Method

### 2.1 Setup

A schematic of the experimental setup is shown in Fig. 1(b). The output beam from a Yb-doped fiber chirped pulse amplification (CPA) laser system,^27^ which produced a 108 fs, 0.37  μJ, 1064 nm pulse at a repetition rate of 7.0 MHz, was separated into two optical paths for an optical manipulation system and a 2P laser scanning microscope (2P-LSM) and recombined by a polarization beam splitter. The laser spectrum range was 1049 to 1072 nm (see Fig. S1 in the Supplementary Material). In the optical manipulation system, a homemade transmission echelle grating^25^ (see Fig. S1 in the Supplementary Material for more details) with a step thickness of 0.55 mm and a step width of 1.02 mm or 0.75 mm was placed conjugate to a digital micromirror device (DMD; Texas Instruments, Dallas, Texas, United States, DLP4500NIR). The DMD acted as a diffraction grating for TF and an amplitude modulator for beam shaping^28^^,^^29^ (see Fig. S2 in the Supplementary Material for more details). The beam diameter ($1/e^{2}$ width) on the echelle grating was almost 10 mm. The magnification from the DMD to the echelle grating was 4.0. The echelle grating and the DMD were positioned at the focal plane of a water-immersion objective lens (OB1; Nikon, Minato, Japan, MRD77225, 25×) with an NA of 1.1 through 4f optical systems at a magnification of 1/750 and 1/187.5, respectively. The electric field on the DMD was Fourier-transformed by a lens with a focal length of 1000 mm into an LCOS-SLM (Hamamatsu Photonics, Hamamatsu, Japan, X15233-08, 1280×1024  pixels, 12.5  μm pixel size, Al mirror type) conjugate to the pupil plane of the objective lens. The LCOS-SLM was used to generate multitargeted spots. By increasing the spot size of a single wavelength on the LCOS-SLM, not only the spectral phase but also the spatial phase was simultaneously modulated so that the spatial focusing position corresponded to the TF position. The imaging magnification from the LCOS-SLM to the pupil was 1.5. To mitigate the thermal effect caused by high-power lasers, a water-cooling system was installed in the LCOS-SLM. To evaluate 2P patterned illumination, 2P excitation fluorescence from a rhodamine B solution excited by the generated patterns was collected by a second objective lens (OB2; Olympus, Shinjuku, Japan, UPLFN10X2, 10×) with an NA of 0.3 and recorded by a complementary metal oxide semiconductor (CMOS) camera (Hamamatsu Photonics, Hamamatsu, Japan, ORCA-Fusion). The excitation light was then eliminated by a short-pass filter (SPF; Sigma Koki, Tokyo, Japan, SHPF-25C-790). For comparison of TM-ML-TF-CGH with TF-CGH, the TM-ML-TF method was changed to the wide-field TF method by removing the echelle grating.

In the 2P-LSM system, the generated fluorescence was collected by OB1 and reflected by a dichroic mirror (DM; Sigma Koki, Tokyo, Japan, DIMNQ-5172R03-P-R785-R565-T1325) to separate the excitation light. The residual excitation light was eliminated by an SPF and detected with a photomultiplier tube (PMT; Hamamatsu Photonics, Hamamatsu, Japan, H15460-40). Because the 2P-LSM system provides optical sectioning capability without a confocal pinhole, nondescanned detection was employed to increase the detection efficiency of scattered fluorescence.^5^ The x−y cross-sectional 2P fluorescence images of samples were obtained by laser scanning using a combination of a resonant scanner (RS; Cambridge Technology, Boston, Massachusetts, United States, CRS 4 KHz) and a galvanometer scanner (GS; Cambridge Technology, 6230H). The 3D images were acquired by scanning samples mounted onto a stepper motor stage (Suruga Seiki, Shizuoka, Japan, KWC06020-LGC) in the axial direction.

### 2.2 Designing a Transmission Echelle Grating Condition

**Fig. 2:**
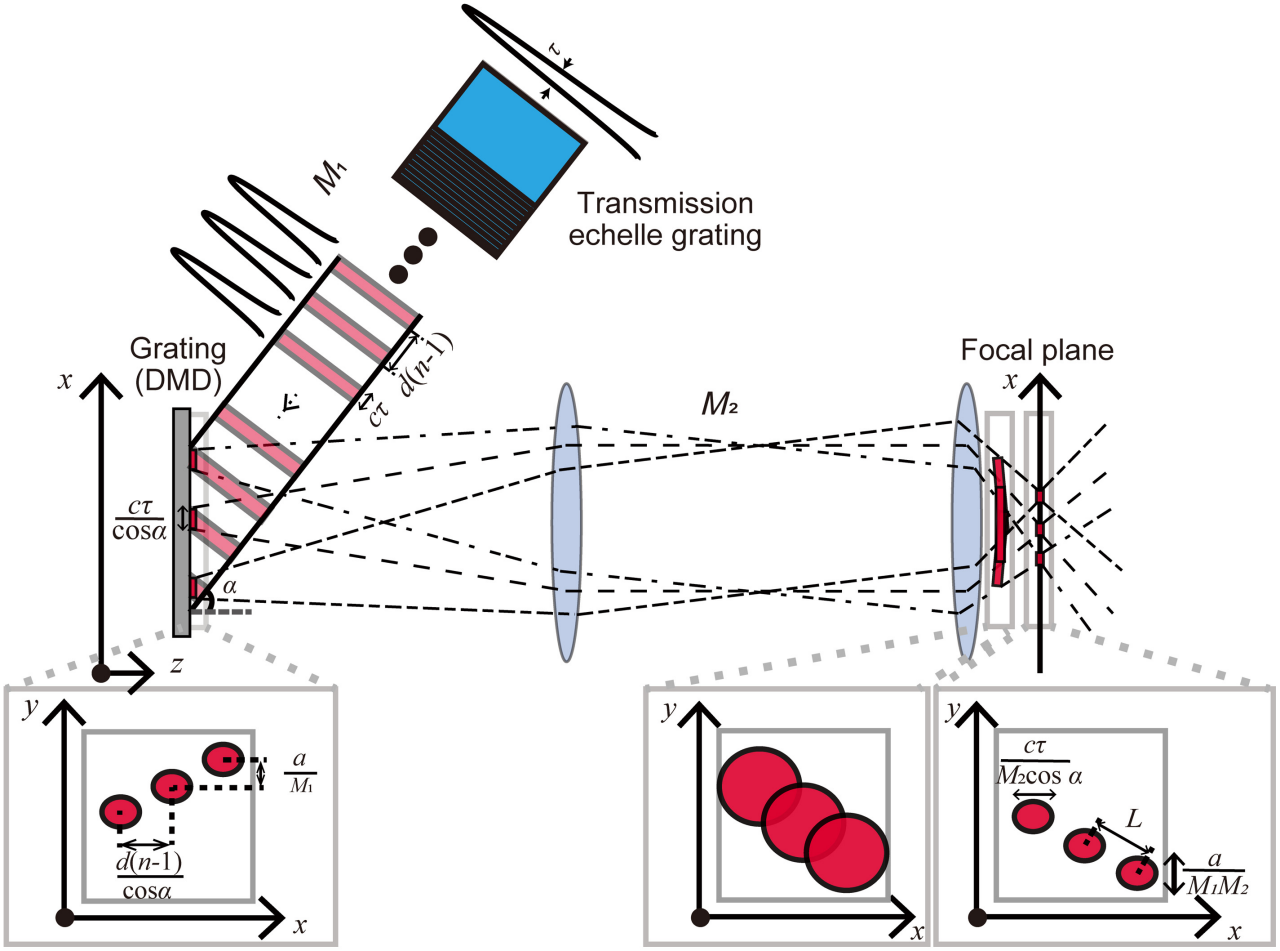
Propagation of multiple transient beams generated by a combination of echelle and diffraction gratings. c is the speed of light in a vacuum. Time-multiplexed pulses from a transmission echelle grating, with a temporal delay of d(n−1)/c, are incident to a grating (DMD) at an incident angle of α. The spacing among the next transient beam at a grating along the x and y directions are d(n−1)/cos α and $a/M_{1}$. The spacing among the next transient beam at the focal plane is reduced by a factor of $M_{2}$. The transient beams away from the focal plane are expanded because of the diffraction.

Now, we consider the use of a transmission echelle grating with a step thickness of d and a step width of a for TM-ML-TF. The echelle grating produces multiple beamlets with a temporal delay among them, which is induced by the difference in refractive index between the glass and air. By increasing the step thickness of the echelle grating, the temporal delay becomes longer than the coherence time τ of the excitation pulses. Under this condition, the beamlets do not interfere with each other near the echelle grating. Because the echelle grating is placed conjugate to the focal plane of an objective lens, each beamlet is diffracted along the y direction and line-focused at each conjugate position of the focal plane of the objective lens. Because a diffraction grating (DMD) for TF is also placed conjugate to the focal plane of an objective lens, the combination of the echelle and diffraction gratings produces multiple transient beams on the focal plane, as shown in Fig. 2.^23^ By increasing the magnification from the focal plane of the objective lens to the echelle grating or reducing the step width of the echelle grating, the spot size of each transient beam along the y direction in the focal plane can be reduced to the size of the diffraction-limited spot. The axial response near the focal plane is determined by the spot size of each transient beam in the focal plane.^23^ If the wavefront is not distorted, the highest axial resolution is achieved under the condition that the demagnified step width of the echelle grating is smaller than the size of the diffraction-limited spot. For the axial response in the out-of-focus region, Talbot images and subimages of the transient beams formed at a certain distance away from the focal plane must be considered.^23^^,^^30^ A spacing among the transient beams on the focal plane is given by^23^

$$L=\sqrt{\{d(n-1)/M_{2}\;\cos\;\alpha {\}}^{2}+(a/M_{1}M_{2}{)}^{2}},$$ (2)

where n is the refractive index of the glass of the echelle grating, α is the incident angle to the diffraction grating, $M_{1}$ is the magnification from the diffraction grating to the echelle grating, and $M_{2}$ is the magnification from the focal plane to the diffraction grating. The axial position of the first Talbot subimage is described as $|z|=L^{2}/2\lambda$, where λ is the central wavelength. If the axial position of the first Talbot subimage is more than approximately two Rayleigh ranges away from the focal plane, the axial response is practically close to that of point focusing.^30^ By increasing the step thickness of the echelle grating or by reducing $M_{1}$ and $M_{2}$, the axial position of the first Talbot subimage can be moved away from the focal plane. However, the demagnified total thickness of the steps of the echelle grating should be smaller than the Rayleigh range.^23^ In addition, large step thicknesses and/or small step widths of the echelle grating increase the effect of partial beam blocking at the edge of the steps. Partial beam blocking causes a stripe pattern at the conjugate position of the echelle grating. However, the stripe pattern on the focal plane can be suppressed by increasing the magnification from the focal plane to the echelle grating so that the demagnified line width of the stripe pattern on the focal plane is smaller than the size of the diffraction spot.

In the experiments, two types of echelle gratings with step widths of 1.02 and 0.75 mm were prepared to compare the beam diameters on the LCOS-SLM. For pattern illumination experiments, the echelle grating with a step width of 0.75 mm was used to achieve high axial resolution. The step thickness of the echelle grating is 0.55 mm, which corresponds to a temporal delay of 848 fs. The temporal delay was much longer than the coherence time of excitation pulses with a duration of 108 fs. $M_{1}$ and $M_{2}$ were 4.0 and 187.5, respectively, and α was 23.1 deg. For the echelle grating with a step width of 0.75 mm, the axial position of the first Talbot subimage was 2.29  μm, which corresponds to approximately two Rayleigh ranges (=2×0.988=1.98  μm).

### 2.3 Evaluation of Thermal Effects of the LCOS-SLM

To evaluate the temporal fluctuation of the modulated phase due to the thermal effects of the LCOS-SLM, we generated first-order diffraction light by applying a blazed grating phase to the LCOS-SLM, which enabled the separation of the first-order diffraction light from the unmodulated zeroth-order light on the focal plane of the objective lens. The temporal fluctuation of the first-order diffraction light and the unmodulated zeroth-order light was recorded by the CMOS camera. The exposure time of the CMOS camera was 80  μs, and the refresh rate was 1.59 kHz. The measurements were performed after sufficient laser irradiation to ensure thermal equilibrium with the LCOS-SLM. In this experiment, we used an echelle grating with a step width of 1.02 mm. A variable neutral-density filter was used behind the LCOS-SLM to prevent saturation of the fluorescence signal with increasing input power. The generated fluorescence intensity for wide-field TF was also adjusted to that for TM-ML-TF using a variable neutral-density filter.

### 2.4 2P Patterned Illumination

The weighted Gerchberg–Saxton (WGS) algorithm was used to create the CGH phase mask for 3D multispot generation.^31^ To increase the maximum lateral and axial displacements without aliasing, the 1280×1024  pixel LCOS-SLM was used instead of the 800×600  pixel LCOS-SLM employed in our previous work (see Figs. S3 and S4 in the Supplementary Material for more details).^26^ If the NA is 1.1, the focal length is 8 mm, the pixel pitch of the LCOS-SLM is 12.5  μm, and the LCOS-SLM has 1024 pixels, the optimized imaging magnification to utilize the NA fully without waste is 1.38. To avoid the effects of scattering at the edges of the LCOS-SLM, the imaging magnification was set to 1.5, which is slightly larger than that of the optimized value. Under this condition, the maximum lateral and axial displacements without aliasing were calculated to be ±226 and ±155  μm, respectively.

To characterize 2P patterned illumination, the x−y cross-sectional 2P fluorescence images from a thin layer of a rhodamine B solution excited with patterned illumination through OB1 (NA 1.1) were collected by OB2 (NA 0.3) and recorded by the CMOS camera. To prevent the rhodamine B solution from drying out, rhodamine B in a viscous solution was prepared for the sample. A solution of rhodamine B in ethanol (1 mM) was diluted in glycerin to achieve a rhodamine B concentration of 500  μM in a 50% glycerin-ethanol mixture. Then, 0.2  μL of the rhodamine B mixed solution was pipetted onto a glass slide and covered with glass (18×18  mm, No. 1, Matsunami Glass). We also confirmed that the axial response measured using the thin layer of the rhodamine B mixed solution was comparable with that measured using a single layer of 200-nm fluorescent beads (Molecular Probes, Eugene, Oregon, United States, F8809) (see Fig. S5 in the Supplementary Material). In this experiment, an echelle grating with a step width of 0.75 mm was used to achieve high axial resolution. The 3D distribution was acquired by scanning OB1 mounted on a piezoelectric transducer (PZT; nPoint, nPFocus100HD) stage in the axial direction with a step of 500 nm relative to OB2. The 2P fluorescence generated by the transient beams was integrated over the exposure time of the CMOS camera placed conjugate to the focal plane of OB2. The exposure time of the CMOS camera was 100  μs.

The measured 2P fluorescence image is described as

$$F(x,y,z)=⨌I_{ex}^{2}(x^{\prime },y^{\prime },z^{\prime },t)S(z-z^{\prime })D(x-x^{\prime },y-y^{\prime },z-z^{\prime })dx^{\prime }\;dy^{\prime }\;dz^{\prime }\;dt,$$ (3)

where S(z) is the axial density distribution of rhodamine B molecules, $I_{ex}(x,y,z,t)$ is the instantaneous excitation intensity distribution, and D(x,y,z) is the point spread function (PSF) of the detection system. If the thickness of the rhodamine B layer is much thinner than the axial response of the excitation and detection PSFs, the measured 2P fluorescence image can be approximated as

$$F(x,y,z)\approx ∭I_{ex}^{2}(x^{\prime },y^{\prime },z,t)D(x-x^{\prime },y-y^{\prime },0)dx^{\prime }\;dy^{\prime }\;dt.$$ (4)

In addition, the excitation lateral spot diameter of 10  μm is much larger than the lateral resolution of the detection system. Therefore, the measured 2P fluorescence image can be regarded as the 2P excitation distribution.

### 2.5 Photoconversion of Spheroids

Spheroids were cultured from living mouse mammary epithelial EpH4 cells engineered to express PSmOrange2. EpH4 cells were cultured spherically in three dimensions for ∼8 to 12 days using Matrigel (Corning, Corning, New York, United States, #356237). EpH4 cells (American Type Culture Collection) were cultured at 37°C and 5% ${\mathrm{CO}}_{2}$ in Dulbecco’s Modified Eagle Medium (Nacalai Tesque, Kyoto, Japan) supplemented with 10% fetal bovine serum (Thermo Fisher Scientific, Waltham, Massachusetts, United States, Hyclone) and 100  units/mL penicillin and 100  mg/mL of streptomycin (Nacalai Tesque, Kyoto, Japan).^32^ PSmOrange2 is a monomeric fluorescent protein that is photoconvertible from orange to red fluorescence.^33^ Nucleus-localized PSmOrange2 cDNA was amplified by PCR from the pH2B-PSmOrange2 plasmid (Addgene, Watertown, Massachusetts, United States, #34960) and subcloned into the expression vector. Before patterned illumination, the 3D fluorescence image of the spheroids was acquired with the 2P-LSM system at an excitation wavelength of 1060 nm for the orange form of PSmOrange2. Because PSmOrange2 can be photoconverted at a two-photon excitation wavelength of 1060 nm, sufficiently low laser power (3.3 mW) was used for imaging to avoid the photoconversion of PSmOrange2. The x−y cross-sectional 2P fluorescence images were obtained by averaging 20 fluorescence images acquired at a rate of 15 Hz. The z-stack images were acquired with an axial step of 1  μm. The multitarget cells were selected from the 3D fluorescence image of the spheroids. The patterned light generated by the CGH phase mask was irradiated to the multitarget cells at 55.1 mW and photoconverted from orange to red. The orange fluorescence signals were monitored with the CMOS camera, whereas the patterned light was irradiated. The patterned illumination was stopped when the orange fluorescence signal became weak because of photoconversion. The irradiation time was 383 s. After patterned illumination, the 3D fluorescence image of the spheroids was again acquired using the 2P-LSM system under the same condition as before the patterned illumination. By comparing the orange fluorescence images before and after the patterned illumination, we evaluated the photobleaching regions of orange form due to orange-to-red photoconversion.

## 3 Results

### 3.1 Thermal Effects of the LCOS-SLM

**Fig. 3:**
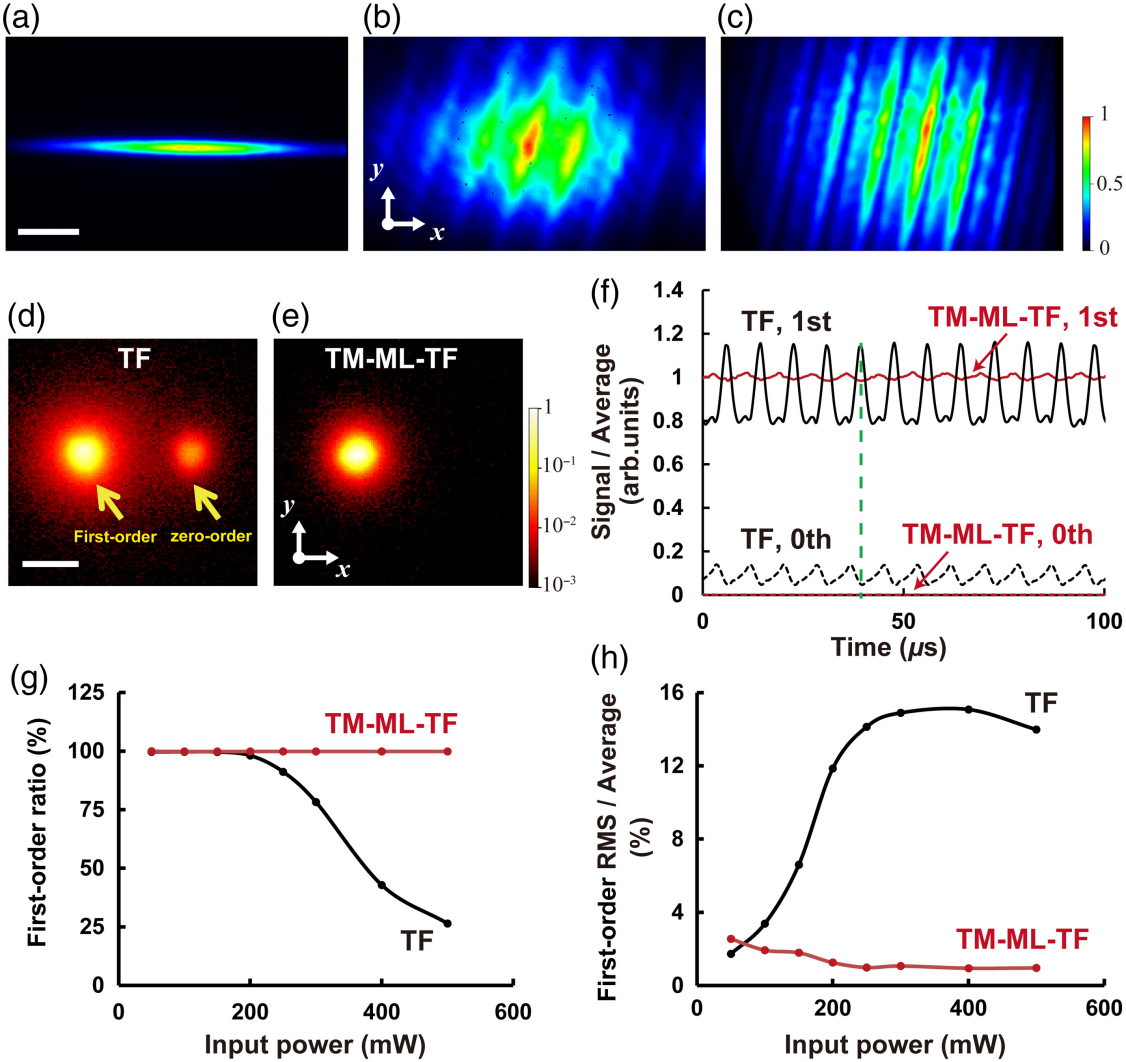
(a)–(c) Beam profiles on the LCOS-SLM placed conjugate to the pupil plane of OB1 for (a) wide-field TF and (b) and (c) TM-ML-TF using the echelle grating with a step width of (b) 1.02 mm and (c) 0.75 mm. The scale bar is 2 mm. (d) and (e) 2P fluorescence images (log-scale) obtained by focusing the first-order diffraction light and the unmodulated zeroth-order light with OB1 for (d) wide-field TF and (e) TM-ML-TF using the echelle grating with a step width of 1.02 mm. The scale bar is 20  μm. (f) Time fluctuation of zeroth-order (dotted line) and first-order (line) 2P fluorescence signals by wide-field TF (black) and TM-ML-TF (red) at 250 mW on the LCOS-SLM. (g) First-order signal ratio for each input power of TF and TM-ML-TF. (h) First-order RMS of the time fluctuation for each input power of wide-field TF and TM-ML-TF.

We first measured the beam profile on the LCOS-SLM placed conjugate to the pupil plane of OB1. As shown in Fig. 3(a), the beam shape for wide-field TF was linear, with a width of 8.27 mm in the x direction and 0.69 mm in the y direction. By contrast, in TM-ML-TF using the echelle grating with a step width of 1.02 mm or 0.75 mm, the illumination beam was expanded to a diameter of 5.8 and 10.4 mm in the y direction, respectively [Figs. 3(b) and 3(c)]. We confirmed that the beam diameter on the LCOS-SLM can be expanded by decreasing the step width of the echelle grating. We also found the tilted stripe patterns in Figs. 3(b) and 3(c). Because first-order diffracted light in the x direction is generated from the DMD and multiple higher order diffracted lights in the y direction are generated from the echelle grating, the combination of the DMD and the echelle grating produces a 2D spectral dispersion on the LCOS-SLM.^24^ The stripe patterns indicate spectral interference and higher spectral resolution of 2D spectral dispersion (see Fig. S1 in the Supplementary Material for more details). Because fringes were observed in the spectrum of the CPA laser system compared with that of the oscillator (see Fig. S1 in the Supplementary Material), spectral interference due to self-phase modulation in the Yb-doped fiber CPA laser system was also visualized as the stripe pattern.

Next, we measured the 2P fluorescence intensity generated by focusing the first-order diffraction light and the unmodulated zeroth-order light with OB1. Figures 3(d) and 3(e) show the 2P fluorescence distributions on a logarithmic scale at an average power of 250 mW on the LCOS-SLM by wide-field TF and TM-ML-TF, respectively. In wide-field TF, not only the first-order but also the zeroth-order fluorescence was observed because of the thermal effect of the LCOS-SLM. On the contrary, the zeroth-order fluorescence was dramatically suppressed by TM-ML-TF. Figure 3(f) indicates the temporal fluctuation obtained by the ratio of the instantaneous zeroth- and first-order fluorescence to the sum of time-averaged zeroth-order and first-order fluorescence. Although the AC drive frequency varies by manufacturer, the frequency of the LCOS-SLM used in the present study was ∼120  Hz. Thus, the modulated phase varied at a frequency of 120 Hz. The zeroth- and first-order fluorescence of wide-field TF fluctuated substantially over time at this frequency. As can be seen from the signal at the time indicated by the green dotted line in Fig. 3(f), the phase of fluctuation of the first-order fluorescence of wide-field TF was opposite to that of the zeroth-order fluorescence. In addition, the sum of instantaneous zeroth-order and first-order fluorescence was not equal to the sum of time-averaged zeroth-order and first-order fluorescence. This is because the 2P fluorescence intensity is proportional to the square of the excitation intensity. These results indicate that the energy of the first-order light was transferred to that of the zeroth-order light. Therefore, we concluded that the phase modulated by the LCOS-SLM varied periodically because of the thermal effect caused by the line focusing on the LCOS-SLM. By contrast, the temporal fluctuation of the first-order fluorescence by TM-ML-TF could be dramatically suppressed, as could the zeroth-order fluorescence because the beam diameter was expanded on the LCOS-SLM. Figures 3(g) and 3(h) show the ratio of the time-averaged first-order fluorescence intensity to the time-averaged total fluorescence intensity and the ratio of the root mean square (RMS) of the first-order fluorescence intensity to the time-averaged first-order fluorescence intensity, respectively. In the case of wide-field TF, when the average input power on the LCOS-SLM exceeded 200 mW, the ratio of the first-order fluorescence intensity decreased rapidly and the temporal fluctuation surpassed 10%. Because the proportion of unmodulated zeroth-order light increased as a result of the thermal effect of high input power, the light utilization efficiency decreased. We found that, even if the unmodulated zeroth-order light was blocked, the temporal fluctuation of the first-order diffraction light remained. Conversely, in the case of TM-ML-TF, the ratio of the first-order fluorescence intensity changed little [Fig. 3(g)], and the temporal fluctuation was insignificant, even when the average input power of the LCOS-SLM was 500 mW [Fig. 3(h)]. These results demonstrate the importance of reducing the power density of the LCOS-SLM.

### 3.2 Accuracy of the Multispot Pattern

#### 3.2.1 Axial resolution enhancement of single-spot illumination

**Fig. 4:**
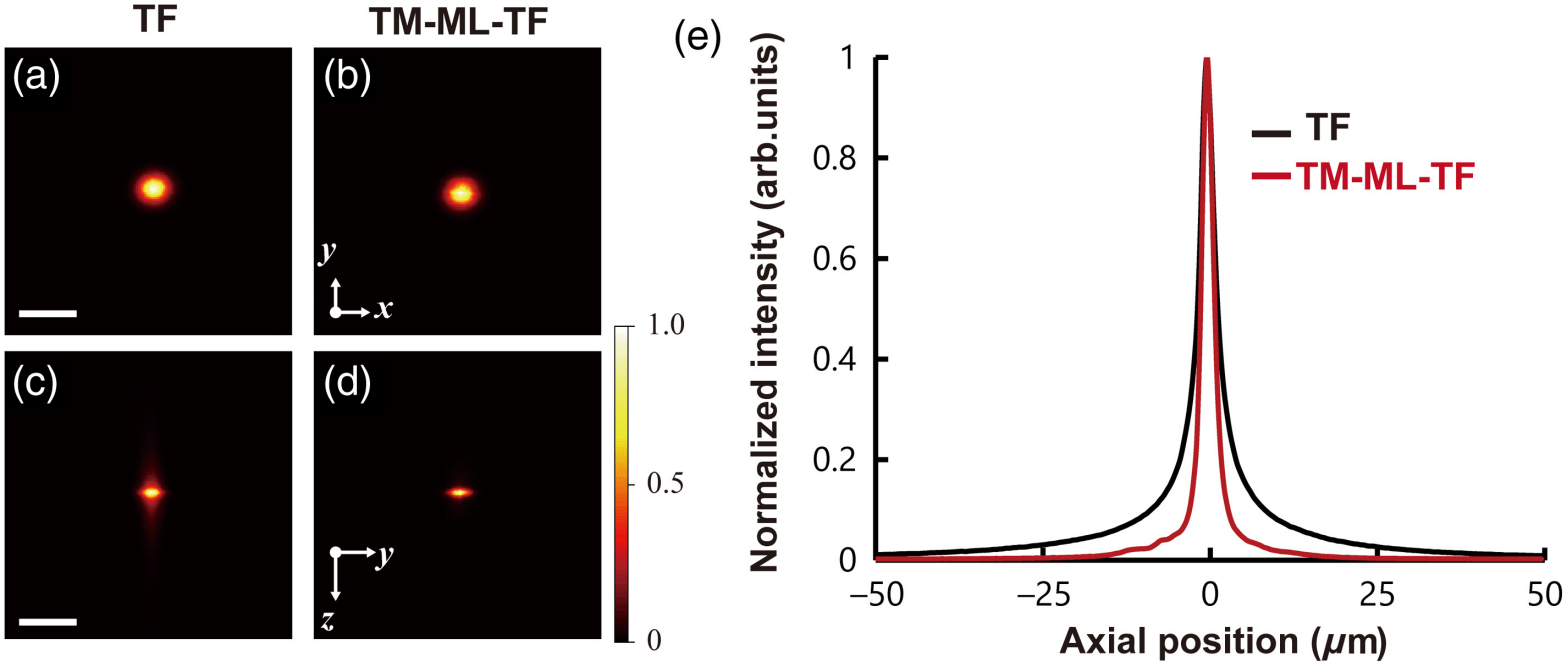
(a)–(d) 2P fluorescence images of a thin rhodamine B solution at the x−y plane, as obtained by (a) wide-field TF and (b) TM-ML-TF, and at the y−z plane, as obtained by (c) wide-field TF and (d) TM-ML-TF. The scale bar is 20  μm. (e) Axial response of 2P excitation for wide-field TF (black line) and TM-ML-TF (red line).

We characterized the axial response of wide-field TF and TM-ML-TF. Figure 4 shows the 2P fluorescence images of a thin layer of rhodamine B solution excited by single-spot illumination with wide-field TF and TM-ML-TF. The lateral spot diameters (${1/e}^{2}$ width) of the 2P fluorescence images for wide-field TF and TM-ML-TF were 10.9 and 10.5  μm in the x direction and 9.2 and 9.4  μm in the y direction, respectively. Figure 4(e) shows the axial response of wide-field TF and TM-ML-TF, as acquired by integrating the fluorescence signal over the x−y direction. The FWHMs for wide-field TF and TM-ML-TF were found to be 2.99 and 2.02  μm, respectively. The ${1/e}^{2}$ widths for wide-field TF and TM-ML-TF were 12.4 and 5.13  μm, respectively. Even the ${1/e}^{2}$ width for TM-ML-TF is smaller than the single-cell size of 10  μm.

#### 3.2.2 Precise lateral multitarget illumination

**Fig. 5:**
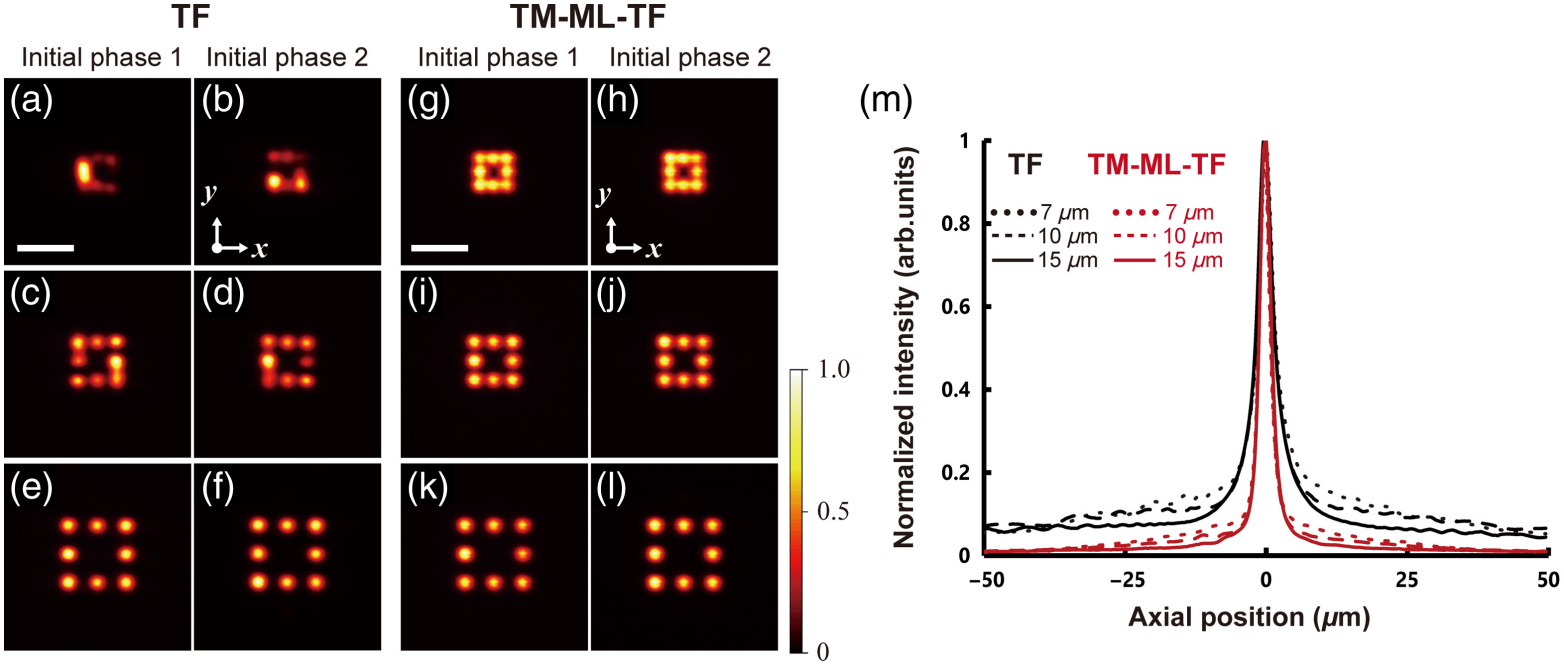
(a)–(l) 2P fluorescence images of a thin rhodamine B solution, as acquired for a laterally arranged eight-spot pattern of (a)–(f) wide-field TF with initial random phase difference (left and second from left) and (g)–(l) TM-ML-TF with an initial random phase difference (right and second from right) at distances of (top) 7  μm, (center) 10  μm, and (bottom) 15  μm. The scale bar is 30  μm. (m) Axial response of 2P excitation at a distance of 7  μm (dotted line), 10  μm (dash-dotted line), and 15  μm (solid line), with initial phase 1 for wide-field TF (black) and TM-ML-TF (red).

To demonstrate precise lateral multitarget illumination with minimal interference among spots, we generated multiple spots laterally in close proximity. We set the phase mask on the LCOS-SLM to arrange eight spots on the focal plane of OB1. The average power at the sample was 38 mW. Figures 5(a)–5(l) show the 2P fluorescence images of the eight spots with between-spot distances of 7, 10, and 15  μm. At a distance of 7  μm, the pattern generated using wide-field TF differed from the designed pattern because the spatiotemporal overlap of the spots along the direction perpendicular to the spectrally dispersed direction caused interference among them [Figs. 5(a) and 5(b)]. This problem does not occur in holographic beam shaping using phase modulation because the CGH phase masks are iteratively designed to produce the desired overall 2D pattern, including light-free areas across the focal plane.^16^^,^^17^ In addition, alternating the initial random phase of each spot to prepare the CGH mask led to corresponding changes in the interference pattern, thereby complicating the generation of a precisely designed pattern. The wide-field TF pattern at a distance of 10  μm, which was comparable with the spot diameter, appeared to be the same as the designed pattern [Figs. 5(c) and 5(d)]. However, the intensity of each spot was strongly dependent on the initial random phase. The intensity of each spot in the wide-field TF pattern at a distance of 15  μm, which was greater than the spot diameter, was independent of the initial random phase [Figs. 5(e) and 5(f)]. By contrast, TM-ML-TF provided the same pattern as the design pattern irrespective of the initial random phase [Figs. 5(g)–5(l)] because interference among the spots could be suppressed by not only the temporal delay along the direction parallel to the direction of spectral dispersion introduced by the diffraction grating for TF but also that perpendicular to the direction of spectral dispersion introduced by the echelle grating. Figure 5(m) shows the 2P excited fluorescence intensity distribution along the axial direction in Figs. 5(a), 5(c), 5(e), 5(g), 5(i), and 5(k). As the distance among multiple spots decreases, the probability of 2P excitation in the out-of-focus region increases. At an axial distance of 10  μm from the focal plane, the fluorescence intensity for TM-ML-TF at a distance of 7  μm was smaller than that for wide-field TF at a distance of 15  μm.

#### 3.2.3 Precise axial multitarget illumination

**Fig. 6:**
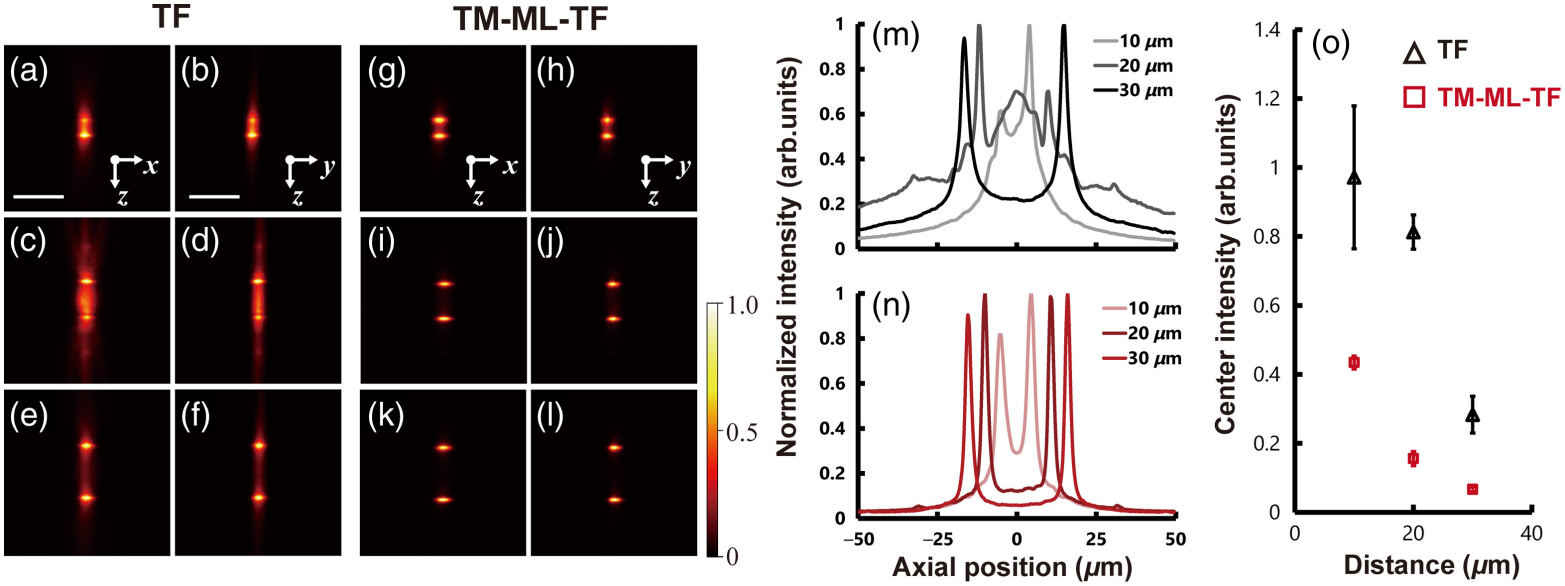
2P fluorescence x−z and y−z images and the axial response of a thin rhodamine B layer by axially arranged two-spot pattern with (a)–(f) and (m) wide-field TF and (g)–(l) and (n) TM-ML-TF at a distance of 10, 20, and 30  μm, respectively. The scale bar is 30  μm. (m) and (n) Axial distribution of the integrated 2P fluorescence intensity over the x−y direction of the axial multispot shown in (a)–(l). (o) The center intensity between the two spots at each distance with wide-field TF and TM-ML-TF.

We next demonstrate precise axial multitarget illumination with TM-ML-TF. We designed the CGH phase mask of LCOS-SLM for two spot patterns with between-spot distances of 10, 20, and 30  μm along the axial direction. Figures 6(a)–6(l) show the x−z and y−z cross-sectional images of the 2P patterned illumination with the CGH phase mask containing the initial random phase, which provides the best of wide-field TF pattern illumination. Figures 6(m) and 6(n) show the axial distributions acquired by integrating the wide-field TF and TM-ML-TF fluorescence signals in Figs. 6(a)–6(l) over the x−y direction. In the wide-field TF pattern, the two spots with a distance of 10  μm could barely be resolved. In imaging, spatial resolution is determined once the two spots are resolved. Conversely, in photostimulation, the intensity at the midpoint between the two spots must be sufficiently low to avoid unintended photostimulation. Although a distance of 20  μm far exceeded the FWHM of the axial resolution, the two spots with a distance of 20  μm strongly interfered in the axial direction; in addition, enhanced fluorescence is observed at the midpoint between the two spots, likely because, in wide-field TF, the axial tail of a single spot is much longer than the FWHM [Fig. 4(e)]. The wide-field TF pattern at a distance of 30  μm could clearly be resolved into two spots because interference at the midpoint between the two spots was suppressed. By contrast, the TM-ML-TF pattern at a distance of 10  μm was clearly resolved irrespective of distance because of high axial resolution and suppression of interference. Figure 6(o) shows the ratio of the center intensity between the two spots to the average peak intensities of the two spots as a crosstalk artifact among axially separated target spots. The center intensities between the two spots induced by wide-field TF were 97%, 81%, and 28% at a distance of 10, 20, and 30  μm, respectively, whereas the corresponding intensities of the spots induced by TM-ML-TF were 43%, 15%, and 6%, respectively.

#### 3.2.4 Three-dimensional multispot pattern

**Fig. 7:**
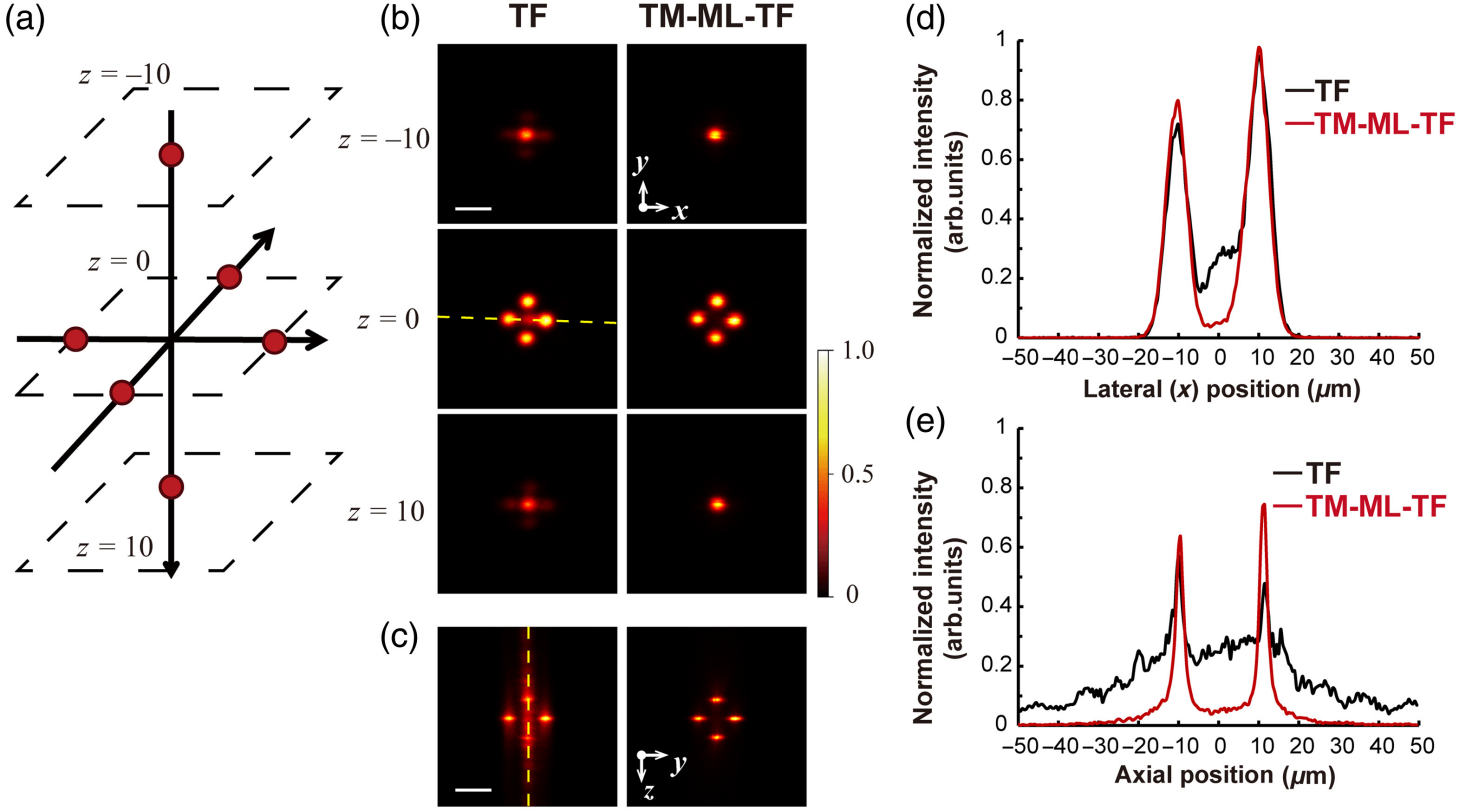
(a) Designed pattern and (b) and (c) 2P fluorescence images of a thin rhodamine B solution excited by the 3D multispot pattern. (left) Wide-field TF and (right) TM-ML-TF in the (b) x−y-plane and (c) y−z-plane. The scale bar is 20  μm. (d) One-dimensional distribution of 2P excitation along the yellow dashed line at z=0  μm shown in panel (b). (d) and (e) 2P intensity distribution along the yellow line shown in panels (b) and (c), as obtained by wide-field TF and TM-ML-TF, respectively.

We evaluated the optical 3D properties of multispot patterned illumination by wide-field TF and TM-ML-TF. Two spots were generated at different z=±10  μm at the same x−y=0  μm, and four spots were generated at the same z=0  μm at different x−y=±10  μm [Fig. 7(a)]. Figures 7(b) and 7(c) show the 2P x−y and y−z cross-sectional images acquired by wide-field TF and TM-ML-TF. Figures 7(d) and 7(e) show the signal distribution along the yellow dotted line in Figs. 7(b) and 7(c), respectively. These results indicate that TM-ML-TF enables the generation of accurate patterns because of high axial resolution and suppression of interference among multispots. We also found that TM-ML-TF can be used to create single cell–size regions intentionally where dark spots occur and activation is avoided.

### 3.3 Field of Excitation (FOE)

**Fig. 8:**
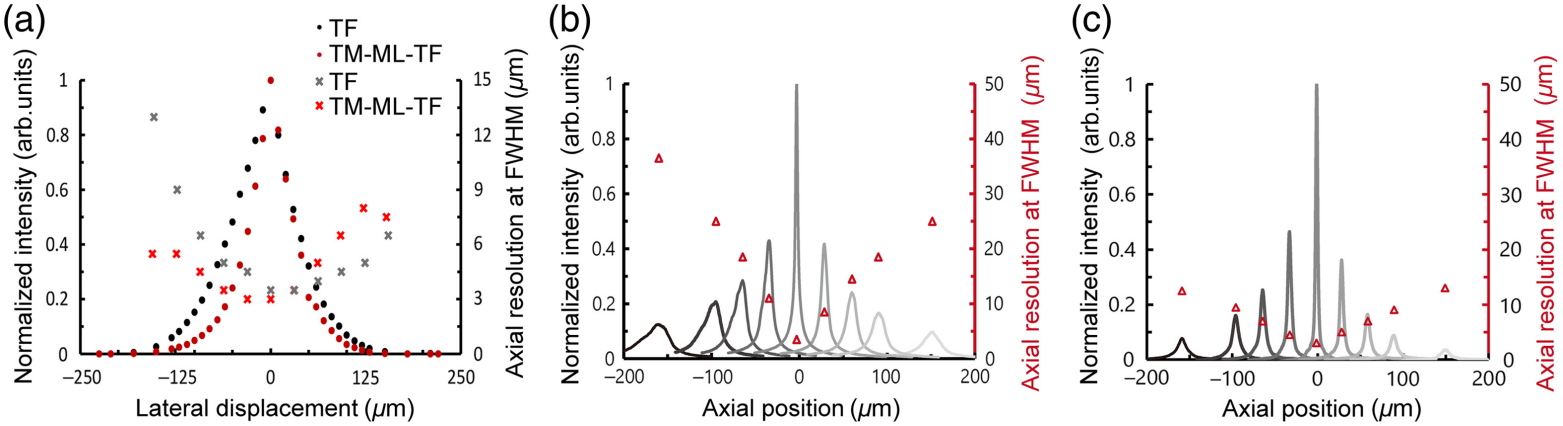
(a) 2P excited fluorescence intensities (•) and axial resolutions (×) at various lateral displacements by TF-CGH (black) and TM-ML-TF-CGH (red). (b) and (c) 2P excited fluorescence intensity profiles (line) and axial resolutions (△) at various axial displacements of −150, −90, −60, −30, 0, 30, 60, 90, and 150  μm by (b) TF-CGH and (c) TM-ML-TF-CGH.

We evaluated the FOE in the lateral direction. Figure 8(a) shows the 2P excited fluorescence intensities and axial resolutions at various lateral displacements by TF-CGH and TM-ML-TF-CGH. Increasing the lateral displacement decreased the 2P excited fluorescence intensity and degraded the axial resolution. This is because increasing the lateral displacement reduces the diffraction efficiency of the LCOS-SLM and increases the aberrations in the optical system^34^ (see more details in the Supplementary Material). At the maximum lateral displacement of ±226  μm, the 2P excited fluorescence intensity was almost 0. Although the 2P excitation intensity variations among multispots can be compensated by the WGS,^16^^,^^17^ the 2P excitation intensity at ±226  μm was too low. Increasing the input laser power increases the 2P excitation intensity, but the thermal effect of the LCOS-SLM limits the input laser power. Thus, the FOE in the lateral direction cannot be defined as twice the maximum lateral displacement without aliasing. According to the ${1/e}^{2}$ widths of the 2P excited fluorescence intensities, the FOE in the lateral direction was evaluated to be almost 200  μm for TF-CGH and 140  μm for TM-ML-TF-CGH. Within the FOE in the lateral direction, the axial resolution was higher than 10  μm.

We also evaluated the FOE in the axial direction. Figures 8(b) and 8(c) show the 2P excited fluorescence intensities and axial resolutions at various axial displacements by TF-CGH and TM-ML-TF-CGH. As the axial displacement increased, the 2P excited fluorescence intensity decreased, and the axial resolution deteriorated. Based on the ${1/e}^{2}$ widths of the 2P excited fluorescence intensities, the FOE in the axial direction was estimated to be 240  μm for TF-CGH and 155  μm for TM-ML-TF-CGH. Compared with TF-CGH, TM-ML-TF-CGH exhibited a narrower FOE in the axial direction, but the axial resolution remained below 10  μm within the FOE. The reduction in 2P excitation intensity at large axial displacements by TM-ML-TF-CGH was greater than that by TF-CGH, whereas the degradation of axial resolution at large axial displacements by TM-ML-TF-CGH was smaller than that by TF-CGH. This is because the laser power loss at the edge of the pupil is larger than that at the center (see more details in the Supplementary Material). Increasing the axial displacement not only reduces the laser power but also reduces the effective NA. In wide-field TF, the laser power loss along the y direction is negligible because the beam shape on the LCOS-SLM is linear along the x direction [see Fig. S7(g) in the Supplementary Material]. However, in TM-ML-TF, the illumination beam on the LCOS-SLM expands along the y direction, resulting in a loss of laser power in the y as well as the x direction [Fig. S7(e) in the Supplementary Material]. Thus, the reduction in two-photon excitation intensity at large axial displacements by TM-ML-TF-CGH is greater than that by TF-CGH. If the effective NA of TM-ML-TF-CGH is the same as that of TF-CGH, the FWHM for TM-ML-TF-CGH is $\sqrt{3}$ times smaller than that for TF. Therefore, in TM-ML-TF-CGH, the degradation of axial resolution due to the decrease in effective NA at large axial displacements was smaller than that in TF-CGH. Increasing the input laser power can compensate for the reduction in two-photon excitation intensity, but not for the degradation in axial resolution.

### 3.4 3D Patterned Photoconversion of Spheroids

**Fig. 9:**
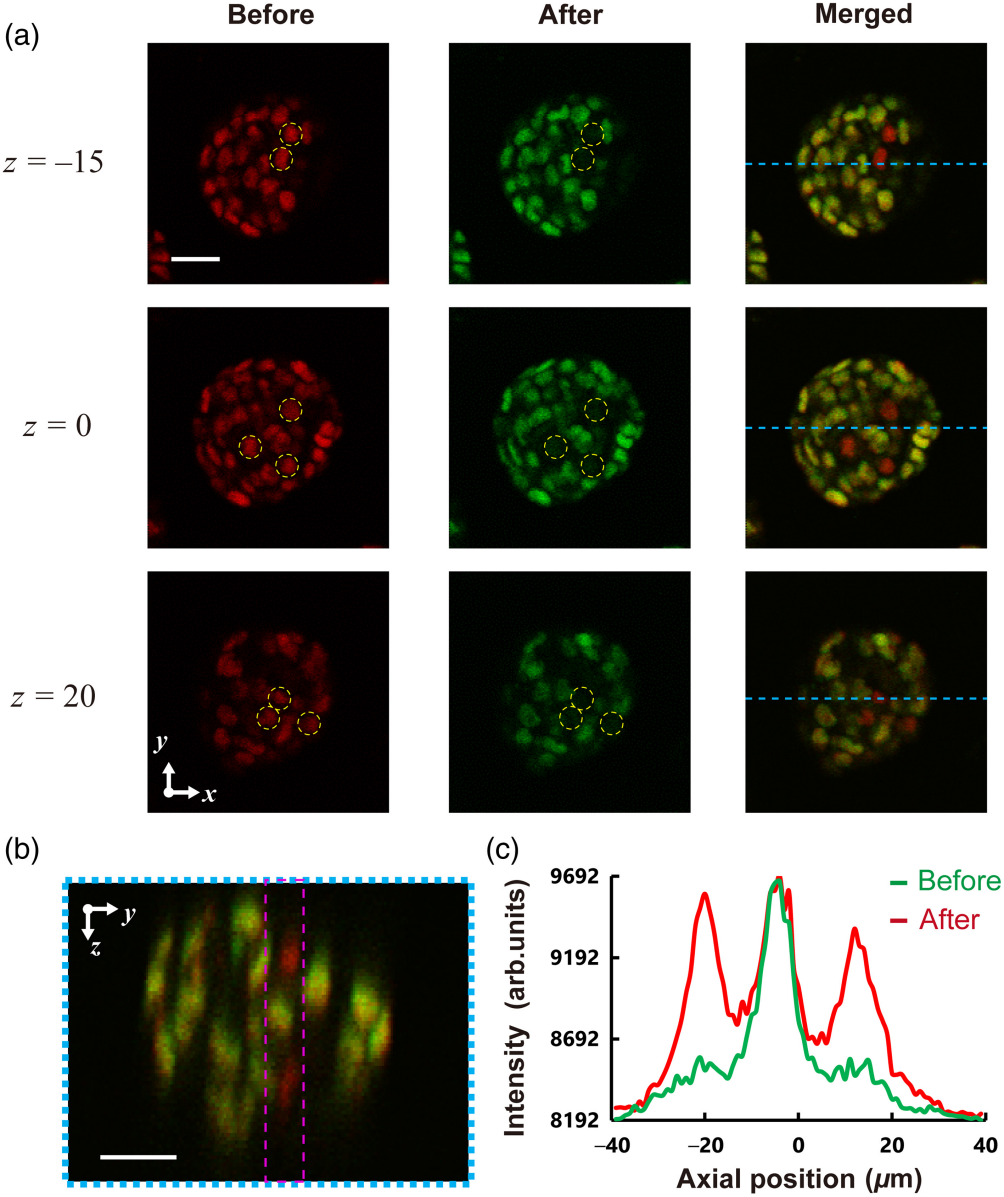
(a) x−y Cross-sectional images of an EpH4-spheroid engineered to express nucleus-localized PSmOrange2 (left) before and (center) after photostimulation of target cells indicated by the yellow dotted circles. (Right) Merged left and center images. (b) x−z Cross-sectional images of merged images at the y-position corresponding to the dashed light-blue line. (c) The 2P fluorescence intensity along the z direction integrated in the x direction within the area enclosed by the dashed pink line in (b). The scale bar is 20  μm.

Finally, we applied TM-ML-TF to photoconvert multitarget cells in spheroids selectively. Figure 9(a) shows the x−y cross-sectional 2P fluorescence images at three axial positions before and after photoconversion using TM-ML-TF and the merged images of the cells in distinct axial planes. A comparison with photoconversion using wide-field TF could not be conducted because of the thermal effects of the LCOS-SLM. The CGH phase mask was produced by targeting the cells indicated by the yellow dotted circles in Fig. 9(a). Figure 9(b) shows the x−z cross-sectional image along the blue dashed line in the merged image in Fig. 9(a). Figure 9(c) indicates the intensity profile along the axial direction integrated in the x direction within the area enclosed by the dashed pink line in Fig. 9(b). We found that the 2P fluorescence intensity of nontarget cells did not change between pre- and post-photoconversion [Fig. 9(c)]. Thus, we concluded that even nontarget cells among target cells along the axial direction were not photoconverted. The multitarget cells were successfully photoconverted, whereas nontarget cells were not. The size of the cell cytoplasm is almost 10  μm, which is larger than the axial ${1/e}^{2}$ width of 5.29  μm of TM-ML-TF, as shown in Fig. 4(e). However, the entire cytoplasm was uniformly photoconverted. Thus, this is due to molecular motion within the cell cytoplasm. These results indicate that patterned illumination with TM-ML-TF can provide 3D single-cell resolution.

## 4 Discussion and Conclusion

We developed a patterned illumination system by combining 3D-CGH and TM-ML-TF techniques. This system solved the problems associated with TF-CGH: thermal effects of the LCOS-SLM, low axial resolution, and interference among multispots.

The effectiveness of the echelle grating for photostimulation has been demonstrated. However, fine-tuning of the echelle grating parameters is remains inadequate. To suppress further the effect of interference among each beamlet at depths away from the focal plane, the axial position of the first Talbot subimage from the focal plane should be further away than two Rayleigh ranges. Although a large step thickness of the echelle grating exacerbates the effect of partial beam blocking at the step edges, increasing the step thickness further extends the axial distance between the first Talbot subimage and the focal plane. The axial distance can also be lengthened by using a medium with a high refractive index.

When the combination of CGH and wide-field TF methods is applied to photostimulation for controlling animal behavior, simultaneous illumination of many neurons is required. As the number of target neurons increases, the average input power increases accordingly. Even when the unmodulated zeroth-order light is blocked to prevent irradiation of undesired areas, the modulated phase varies in time because of the thermal effects of the LCOS-SLM. Thus, stimulating at a constant intensity and with precise timing is difficult. The use of the dielectric multilayer mirror-type LCOS-SLM mitigates this problem. The aluminum-mirror-type LCOS-SLM (Hamamatsu Photonics, Hamamatsu, Japan, X15213-08) has a wide reflection band (1000–1550 nm) because it uses the reflection of light by aluminum pixel electrodes formed on the CMOS chip. However, because aluminum absorbs light, the light utilization efficiency is not so high (82%). The dielectric multilayer mirror-type LCOS-SLM utilizes the reflection of light by the dielectric multilayer film formed on the pixel electrode to achieve high reflectivity and reduce light absorption inside the LCOS chip. Thus, the dielectric multilayer mirror-type LCOS-SLM (Hamamatsu Photonics, Hamamatsu, Japan, X15213-03B) provides higher light utilization efficiency (97%) and weaker thermal effects compared with the aluminum-mirror-type LCOS-SLM; however, the wavelengths range is narrow (1000 to 1100 nm for X15213-03B). The suppression of thermal effects in aluminum-mirror-type LCOS-SLMs using the TM-ML-TF method enables the use of various wavelengths. In the present study, the temporal fluctuation of 2P fluorescence could be substantially reduced from a maximum of 15% to 1%. In addition, the ratio of unmodulated zeroth-order light was reduced by a factor as high as 4.

Photostimulation also necessitates the simultaneous illumination of closely located targets while avoiding the stimulation of untargeted cells. Because the wide-field TF technique causes interference among multispots in close proximity in the direction perpendicular to the spectral direction, the interference pattern changes depending on the initial random phase in the WGS algorithm. This effect complicates the generation of an accurate pattern at the targeted position. As demonstrated in Sec. 3.2.1, the axial resolution in wide-field TF was assessed based on the FWHM of 2.99  μm, which is adequate for resolving individual cells. However, when two spots with an axial distance of 20  μm were generated, the center intensity of the two spots increased to 81%. These results indicate that evaluating the FWHM of the axial intensity is insufficient when generating a multispot pattern. Our proposed combination of CGH and TM-ML-TF can obtain an axial ${1/e}^{2}$ width of 5.29  μm, which is smaller than the single-cell size of 10  μm and can substantially suppress interference due to the temporal delay. These advantages enable single-cell resolution illumination and precise multispot illumination even in close proximity.

The accessible FOE in the lateral direction was smaller than twice the maximum lateral displacement without aliasing because increasing the lateral displacement reduces the diffraction efficiency of the LCOS-SLM and increases the aberrations in the optical system. By compensating the aberrations in the optical system for each spot, the FOE in the lateral direction could be increased up to twice the maximum lateral displacement because the square of the measured diffraction efficiency at the maximum lateral displacement (Fig. S6 in the Supplementary Material) is not so low. Wavefront distortion, caused by the inhomogeneous refractive index distribution of biological tissue, also results in reduced resolution and 2P excitation intensity,^35^ thereby narrowing the FOE and making precise patterned illumination difficult. To improve the performance of patterned illumination in deeper regions, adaptive optics^36^ are needed to correct the wavefront for each spot.

In applications to control animal behavior, photostimulation is needed not only in 3D but also in four-dimensional (4D) space (including time). In our system, the pattern switching speed, which is ∼60  μs, is limited by the LCOS-SLM. Faini et al.^37^ proposed rapidly switching patterns by scanning multiple holograms tiled on an LCOS-SLM. In our configuration, the enlarged spot size on the LCOS-SLM makes using this technique in combination difficult; however, the development of wider LCOS-SLMs is expected to solve these problems. LCOS-SLMs using overdrive with phase-change reduction^38^ could also provide faster 4D photostimulation. Moreover, the ability to change the beam shape depending on the cell is desirable. With our method, although the spot shape can be changed by the DMD, the multispot shape will be uniform. However, multipoint spots can be formed along the cell because interference is less likely to occur. We also consider a method to simplify the implementation of TM-ML-TF. The TM-ML-TF method consists of two 4-f systems: one with an echelle grating for TM-ML and one with a diffraction grating (DMD) for TF. If the combination of a transmission echelle grating and a transmission diffraction grating are integrated as a single optical component, it could be expected to have a simple configuration with only one 4-f system. In conclusion, we demonstrated the effectiveness of 3D CGH with TM-ML-TF, enabling us to localize the axial resolution, suppress the interference among multiple spots, and reduce the thermal effects of the LCOS-SLM. This configuration has the potential to be a powerful tool for the photostimulation of multitarget neurons, opening new possibilities for the future of photostimulation research.

## Supplementary Material

10.1117/1.JBO.30.7.075003.s01

## Data Availability

The datasets used in this paper are available upon reasonable request.
