# Supplementary material for: Three-dimensional scanless patterned illumination using time-multiplexed multiline temporal focusing for multicell manipulation with single-cell resolution
Source: J Biomed Opt. 2025 Jul 28;30(7):075003. doi: 10.1117/1.JBO.30.7.075003 (PMC12302995; doi:10.1117/1.JBO.30.7.075003)
Supplement: Supplementary file 1 [file JBO_030_075003_SD001.pdf]

Supplementary material for

Three-dimensional scanless patterned illumination using time-multiplexed multiline temporal focusing for multicell manipulation with single-cell resolution

Kenta Inazawa,[a,b,c] Mayumi Yamada,[d] Takayuki Michikawa,[a,e,f] Kana Namiki,[e,g] Atsushi Miyawaki,[e,g,h] Itaru Imayoshi,[b,d] Katsumi Midorikawa[a] and Keisuke Isobe[a,i,*]

[a]Attosecond Science Research Team, RIKEN Center for Advanced Photonics, 2-1 Hirosawa, Wako, Saitama 351-0198, Japan
[b]Laboratory of Brain Development and Regeneration, Graduate School of Biostudies, Kyoto University, Kyoto 606-8501, Japan
[c]Electron Tube Division, Hamamatsu Photonics K.K., 314-5 Shimokanzo, Iwata, Shizuoka 438-0193, Japan
[d]Laboratory of Deconstruction of Stem Cells, Institute for Life and Medical Sciences, Kyoto University, Kyoto 606-8501, Japan
[e]Biotechnological Optics Research Team, RIKEN Center for Advanced Photonics, 2-1 Hirosawa, Wako, Saitama 351-0198, Japan
[f]Laboratory of Optical Biomedical Science, Kyoto University, Institute for Life and Medical Sciences, Kyoto 606-8501, Japan
[g]Laboratory for Cell Function Dynamics, RIKEN Center for Brain Science, 2-1 Hirosawa, Wako, Saitama 351-0198, Japan
[h]Laboratory of Bioresponse Analysis, Institute for Life and Medical Sciences, Kyoto University, Kyoto 606-8507, Japan.
[i]Laboratory of Spatiotemporal Optical Control, Graduate School of Biostudies, Kyoto University, Kyoto 606-8501, Japan
*Address all correspondence to Keisuke Isobe, kisobe@riken.jp

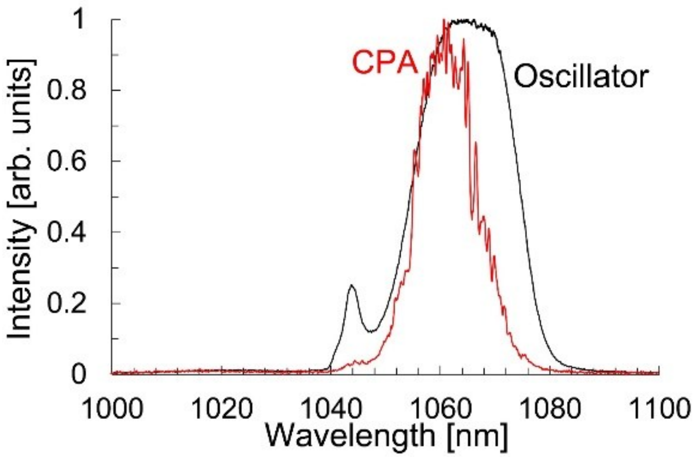

Figure S1. Output spectra from a Yb-doped fiber oscillator and a Yb-doped fiber chirped pulse amplification (CPA) laser system. Compared with the spectrum of the oscillator, that of the CPA laser system shows fringes because of spectral interference due to self-phase modulation.

S1 Homemade transmission echelle grating

The transmission echelle grating was fabricated by stacking 550-μm thick borosilicate cover glasses (22 mm × 40 mm, No. 5, Matsunami Glass) as shown in Fig. S2(a). A shim plate (thickness: 50 μm) was placed between the two cover glasses because anti-reflection coatings were applied to both surfaces of the cover glass. The positions of the cover glasses were aligned with the mold made of aluminum.

We now consider the diffraction condition of the homemade transmission echelle grating. When the incident light is incident to the cover glass perpendicularly, the angle θ between the diffraction light and incident light is expressed as

$$nd + s - \sqrt{a^2 + (d+s)^2}\sin\theta = m\lambda.$$

Here d and n are the thickness and refractive index of the cover glass, respectively, a is the step width of the echelle grating, s is the thickness of the shim plate, m is the diffraction order, and λ is the wavelength. Considering the blaze condition to maximize the diffraction efficiency, the angle θ must be 0°. Thus, the blaze wavelength is given by

$$\lambda_{blaze} = \frac{nd + s}{m}.$$

Figures S2(b) and (c) show the calculated blaze wavelengths and diffraction angles at various diffraction orders for $n = 1.51$, $d = 550$ μm, $s = 50$ μm, and $a = 750$ μm.

The diffracted lights were collimated by a lens L1 with a focal length of 1000 mm and focused on a digital micromirror device (DMD) by a lens L2 with a focal length of 250 mm. The diffracted lights from the DMD were collimated by a lens L3 with a focal length of 1000 mm and impinged on a liquid-crystal-on-silicon spatial light modulator (LCOS-SLM). The diffracted lights on the LCOS-SLM were projected on the pupil plane of the objective lens (OB1; Nikon, MRD77225,

25×) in the second 4-f optical system with a magnification of 1.5. Because the pupil diameter of the objective lens is 17.6 mm, the acceptable diffraction angle of the echelle grating entering the pupil is nearly 0.08°. Because the laser spectrum range was 1049 nm to 1072 nm, the multiple higher-order diffracted lights from the 820th to 839th order are irradiated onto the sample. Adjacent orders of diffracted light in the y direction from the echelle grating can be separated by generating first-order diffracted light in the x direction from the DMD as a diffraction grating. Because the wavelength on the horizontal axis of Fig. S2(c) can be regarded as the diffraction angle of the DMD, the combination of the DMD and the echelle grating produces a two-dimensional (2D) spectral dispersion. If the spot diameter at a single wavelength in the 2D spectral dispersion is smaller than the space between adjacent orders of diffracted light, the tilted stripe pattern appears at the Fourier planes of the diffraction grating and echelle grating. The tilted stripe pattern indicates higher spectral resolution. Because the spectral resolution of 2D spectral dispersion is higher than that of one-dimensional spectral dispersion, finer stripe patterns resulting from spectral interference can be observed.

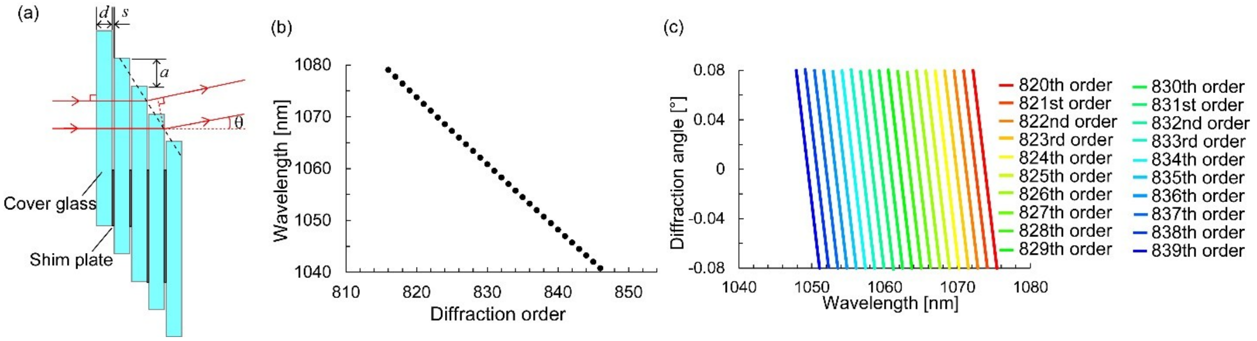

Figure S2. (a) Schematics for homemade transmission grating with a step thickness *d*, a thickness of a shim plate, and a step width *a*. The diffraction angle of the light through a transmission grating is θ. (b, c) The blaze wavelengths (b) and diffraction angles (c) at various diffraction orders for n = 1.51, d = 550 µm, s = 50 µm, and a = 750 µm.

S2 Digital micromirror device

As a diffraction grating for temporal focusing (TF) and an amplitude modulator for beam shaping, the DMD (Texas Instruments, DLP4500NIR, 912×1140 resolution diagonal array) with a diagonal micromirror pitch of 7.6 μm, a horizontal micromirror pitch of 10.8 μm and a micromirror tilt angle of ±12° (Fig. S3(a)) was used. The tilt angle θ_b of the micromirror is regarded as the blaze angle of a blazed grating (Fig. S3(b)). The blaze condition is given by

$$\theta_i = 2\theta_b - \theta_d,$$

where θ_i and θ_d are angles of incidence and diffraction, respectively. To match the TF and the multiline focusing planes, the diffraction angle of the central wavelength must be zero. Thus, to maximize the diffraction efficiency, the angle of incidence should be nearly 24°. In addition, the incidence and diffraction angles must satisfy the diffraction condition:

$$L(\sin\theta_i + \sin\theta_d) = m\lambda,$$

where L is the horizontal micromirror pitch, m is the diffraction order, and λ is the wavelength. Considering the blaze and diffraction conditions for L = 10.8 μm and λ = 1.06 μm, a fourth-order diffraction light at an angle of incidence of 23.1° was used. Under this condition, the angular dispersion was calculated to be 0.0212 degrees/nm.

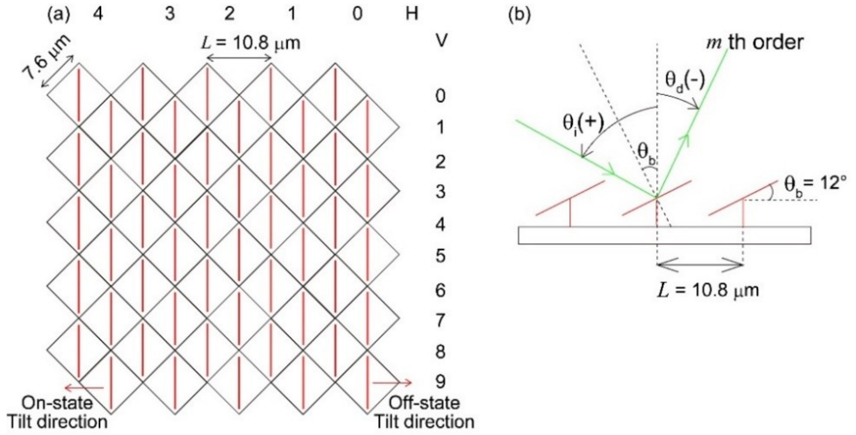

Figure S3. (a) Micromirror array, pitch, and hinge-axis orientation. (b) The DMD acted as a blazed grating.

The DMD functions as an amplitude modulator when some micromirrors are turned off. We investigated the effects of angular dispersion due to amplitude modulation for beam shaping by DMD in TF. Figure S4 shows the beam profiles with amplitude modulation, which were measured on the LCOS-SLM placed conjugate to the pupil plane of OB1. A smaller number of turn-on pixels in the y direction (Fig. S4(a)) leads to a wider beam diameter in the y direction as a result of diffraction of the Gaussian beams on the DMD (Fig. S4(b)). Reducing the number of turn-on pixels in the x direction (Fig. S4(c)) does not seem to make a difference (Fig. S4(d)). This is because the diffraction direction of each monochromatic beam coincides with the diffraction direction as a blazed diffraction grating, resulting in spatial and spectral overlap. Consequently, the spectral resolution at the pupil plane was degraded. These results also indicate that the angular dispersion remains constant.

We created a pentagram-shaped pattern to evaluate the axial confinement characteristics with amplitude modulation. The pentagram consisted of radius of 100 pixels. Figures S4(e) and (f) show the two-photon (2P) fluorescence image by TF and TM-ML-TF with a pentagram-shaped pattern. As shown in Fig. S4(g), the axial response of TF with a pentagram-shaped pattern is almost the same as that of TF without a pentagram-shaped pattern.

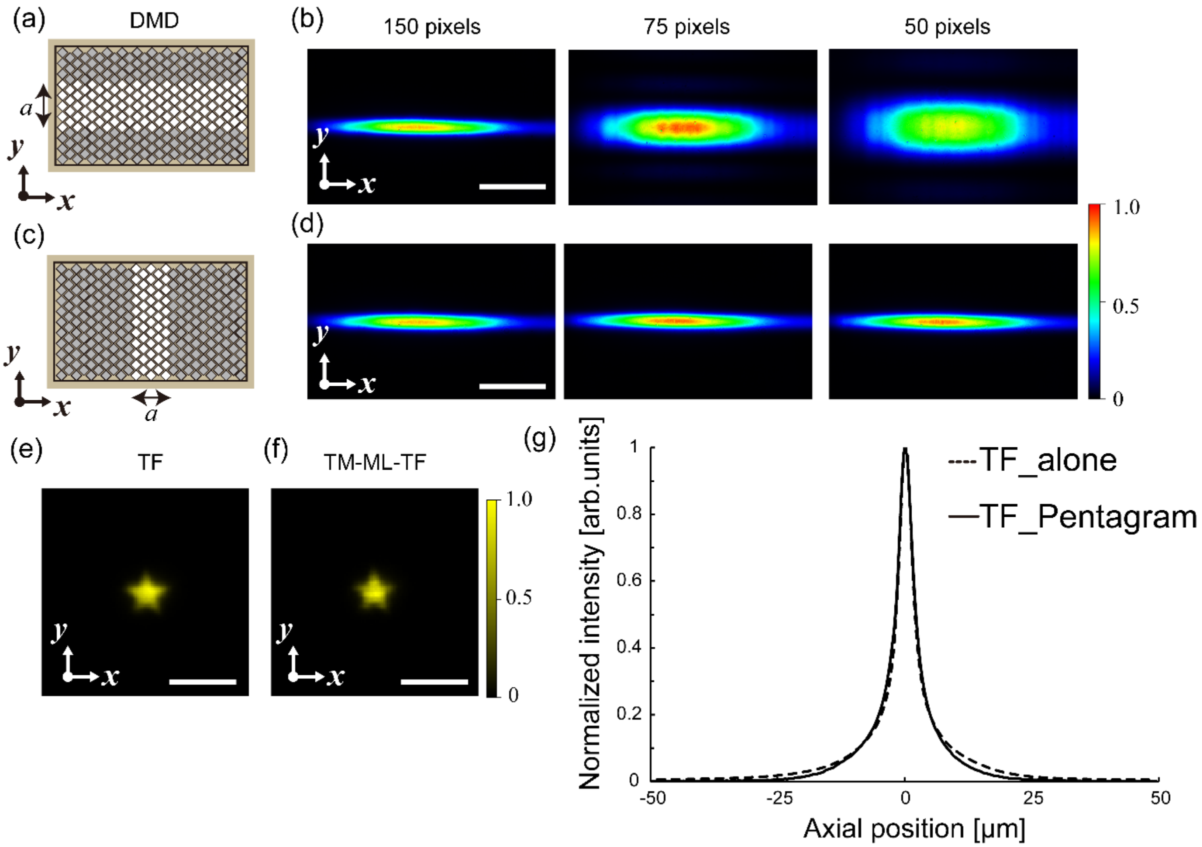

Figure S4. Effect of angular dispersion due to amplitude modulation for beam shaping by DMD in TF. (a) A schematic of some micromirrors in the *y* direction are turned on for beam shaping. (b) Beam profiles on the LCOS-SLM when the micromirrors with 150 (left), 75 (center), and 50 (right) pixels in the *y* direction are turned on. Scale bar is 30 μm. (c) A schematic of some micromirrors in the *x* direction are turned on for beam shaping. (d) Beam profiles on the LCOS-SLM when the micromirrors with 150 (left), 75 (center), and 50 (right) pixels in the *x* direction are turned on. Scale bar is 30 μm. (e, f) 2P fluorescence image by TF (e) and TM-ML-TF (f) with a pentagram-shaped pattern. Scale bar is 10 μm. (g) Axial responses of TF without (dashed line) and with (line) a pentagram shaped pattern.

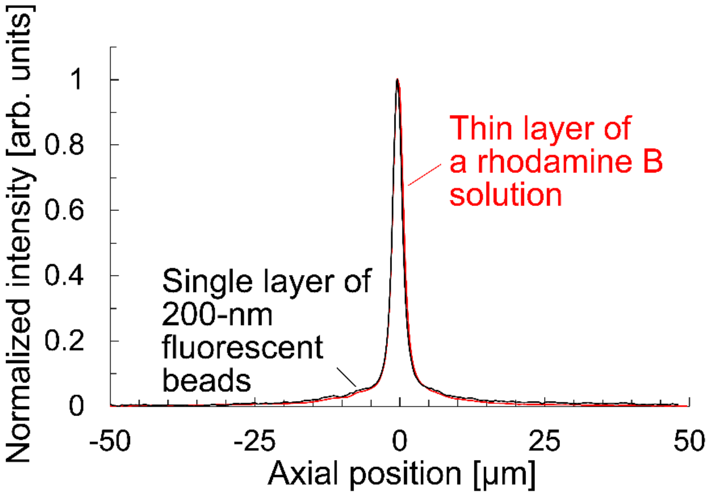

Figure S5. Axial responses measured using the thin layer of the rhodamine B mixed solution (red) and a single layer of 200-nm fluorescent beads (Molecular Probes, F8809) (black).

S3 Field of excitation in the lateral direction

The phase distribution for the target lateral displacement (Δx, Δy) is expressed as

$$\phi_{xy}(x,y) = \frac{Mk_0}{f_{OB}}\left(\Delta x x + \Delta y y\right),$$

where M is the imaging magnification from the LCOS-SLM to the pupil of the objective lens, k_0 is the wavenumber in vacuum, and f_{OB} is the focal length of the objective lens. To satisfy the aliasing-free condition, the phase difference between adjacent pixels on the LCOS-SLM must be less than π:

$$w\frac{\partial \phi_{xy}(x,y)}{\partial x} = \frac{Mwk_0\Delta x}{f_{OB}} \le \pi,$$

where w is the pixel pitch of the LCOS-SLM. Thus, the maximum lateral displacement, $\Delta x_{\max}$, is given by

$$\Delta x_{\max} = \frac{\lambda f_{OB}}{2Mw},$$

where λ is the wavelength. The maximum lateral displacement is inversely proportional to the imaging magnification from the LCOS-SLM to the pupil. However, the smaller imaging magnification results in low NA. To fulfill the pupil without waste, the diameter of the LCOS-SLM, $D_{\mathrm{SLM}} = wN_{SLM}$, must be magnified to meet the pupil diameter, $D_{\mathrm{pupil}} = 2NAf_{OB}$, i.e., $M = D_{\mathrm{pupil}} / D_{\mathrm{SLM}}$. Here, NA is the numerical aperture of the objective lens and N_{SLM} is the number of pixels of the LCOS-SLM. Under this condition, the maximum lateral displacement is rewritten as

$$\Delta x_{\max} = \frac{\lambda N_{SLM}}{4NA}.$$

Thus, to increase the maximum lateral displacement, it is important to use LCOS-SLMs with a larger number of pixels. In our previous work (f_{OB} = 3 mm, M = 1.0, w = 20.0 μm, N_{SLM} = 600 pixel, and λ = 1.06 μm),[1] the maximum lateral displacement was 79.5 μm. To extend the maximum

lateral displacement, the 1280×1024 pixel LCOS-SLM was used instead of the 800×600 pixel LCOS-SLM employed in our previous work.[2] For $NA = 1.1$, $f_{OB} = 8$ mm, $w = 12.5$ µm and $N_{SLM} =$ 1024 pixels, the optimized imaging magnification to fulfill the pupil without waste is 1.38. To avoid the effects of scattering at the edges of the LCOS-SLM, the imaging magnification was set to 1.5, which is slightly larger than that of the optimized value. For $f_{OB} = 8$ mm, $M = 1.5$, $w = 12.5$ µm, and $\lambda = 1.06$ µm, the maximum lateral displacement was calculated to be 226 µm. Therefore, the field of excitation (FOE) in the lateral direction, $FOE_{xy} = 2\Delta x_{\max}$, was 452 µm.

To evaluate accessible FOE, the diffraction efficiency of the LCOS-SLM must also be considered. Theoretically, the diffraction efficiency of the LCOS-SLM is given by[3]

$$P = \left[\frac{\sin\left(\dfrac{\pi M w \Delta x}{\lambda f_{OB}} \right)}{\dfrac{\pi M w \Delta x}{\lambda f_{OB}}} \right]^2 \left[\frac{\sin\left(\dfrac{\pi M w \Delta y}{\lambda f_{OB}} \right)}{\dfrac{\pi M w \Delta y}{\lambda f_{OB}}} \right]^2 = \left[\frac{\sin\left(\pi v_x w \right)}{\pi v_x w} \right]^2 \left[\frac{\sin\left(\pi v_y w \right)}{\pi v_y w} \right]^2,$$

where v_x and v_y are the spatial frequencies on the LCOS-SLM along the x and y directions. As shown in Fig. S6, the diffraction efficiency decreases with increasing the lateral displacement (spatial frequency). For the maximum lateral displacement, the diffraction efficiency is calculated to be 40%. Thus, the 2P excitation intensity is reduced to 16%. The measured diffraction efficiency was lower than the theoretical value (Fig. S6). In addition, the 2P fluorescence intensities for TF and TM-ML-TF were lower than the square of the measured diffraction efficiency (Fig. S6); this is due to aberrations in the optical system. Moreover, as the spatial frequency displayed on the SLM increases, unwanted diffraction orders emerge before reaching the Nyquist frequency. The lateral displacement where the unwanted 2P excited fluorescence intensity surpasses that of targeted fluorescence is about 150 µm. Based on these results, the FOE in the lateral direction was estimated to be around 300 µm.

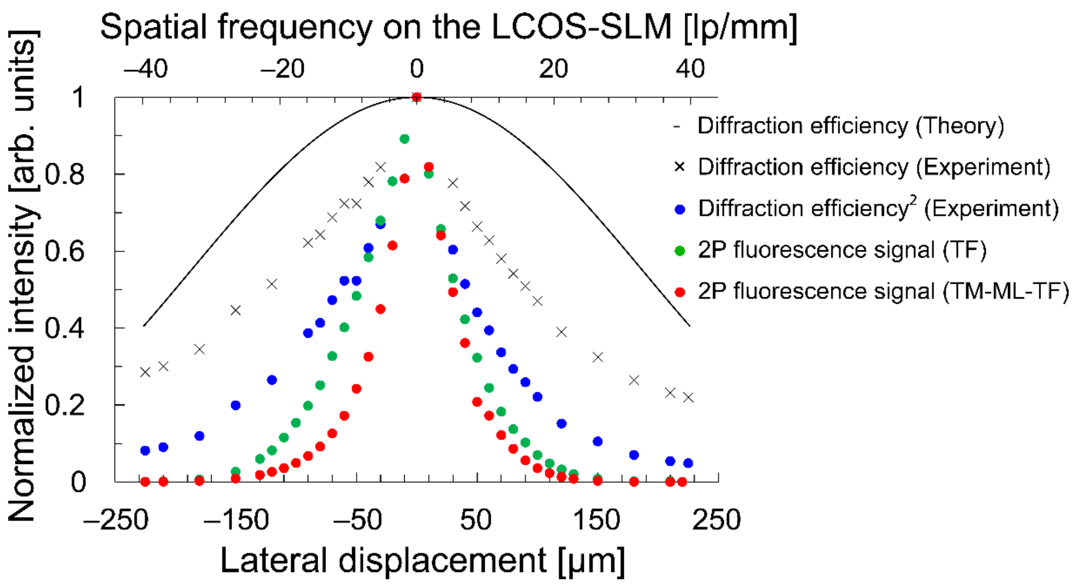

Figure S6. Calculated and measured diffraction efficiencies, and 2P fluorescence intensities for TF and TM-ML-TF at various lateral displacements.

S4 Field of excitation in the axial direction

The phase distribution for the target axial displacement, Δz, is expressed as

$$\phi_z(x,y) = -k_0 \Delta z \left(\sqrt{n_{im}^2 - \frac{M^2 r^2}{f_{OB}^2}} \right),$$

where n_{im} is the refractive index of the immersion medium, $r^2 = x^2 + y^2$. To satisfy the aliasing-free condition, the phase difference between adjacent pixels on the LCOS-SLM must be less than π:

$$w \frac{\partial \phi_z(x,y)}{\partial r} = w \frac{M k_0 \Delta z}{f_{OB}} r \frac{1}{\sqrt{\frac{f_{OB}^2 n_{im}^2}{M^2} - r^2}} \le \pi .$$

The phase difference between adjacent pixels is maximized at the edge of the pupil, $r = NA\, f_{OB} / M$, where NA is the numerical aperture of the objective lens. Thus, the maximum axial displacement, $\Delta z_{\max}$, is given by

$$\Delta z_{\max} = \frac{\pi f_{OB} \sqrt{n_{im}^2 - NA^2}}{k_0 M w NA} = \frac{\lambda f_{OB} \sqrt{n_{im}^2 - NA^2}}{2 M w NA} .$$

For f_{OB} = 8 mm, NA = 1.1, n_{im} = 1.333, M = 1.5, w = 12.5 μm, and λ = 1.06 μm, the maximum axial displacement was calculated to be 155 μm. Therefore, the FOE in the axial direction, FOE_z = $2\Delta z_{\max}$, was 310 μm. If M is assumed to be $M = D_{\text{pupil}} / D_{\text{SLM}}$, then the maximum axial displacement is rewritten as

$$\Delta z_{\max} = \frac{\lambda N_{SLM} \sqrt{n_{im}^2 - NA^2}}{4 NA^2} .$$

Thus, the maximum axial displacement is also proportional to the number of pixels of the LCOS-SLM.

We now consider the local spatial frequencies, (ν_x, ν_y) for $\phi_z(x, y)$ to evaluate the diffraction efficiency. The local spatial frequencies, (ν_x, ν_y) for $\phi_z(x, y)$ are expressed as

$$\nu_x(x,y) = \frac{\dfrac{\partial \phi_z(x,y)}{\partial x}}{2\pi} = \frac{\dfrac{Mk_0\Delta z}{f_{OB}}x}{2\pi\sqrt{\dfrac{f_{OB}^2 n_{im}^2}{M^2} - r^2}},$$

$$\nu_y(x,y) = \frac{\dfrac{\partial \phi_z(x,y)}{\partial y}}{2\pi} = \frac{\dfrac{Mk_0\Delta z}{f_{OB}}y}{2\pi\sqrt{\dfrac{f_{OB}^2 n_{im}^2}{M^2} - r^2}}.$$

As shown in Fig. S7, the local spatial frequency at the edge is higher than that at the center. Because the diffraction efficiency decreases with increasing spatial frequency (Fig. S6), the loss at the edge is larger than that at the center. Thus, increasing the axial displacement reduces not only the laser power, but also the effective NA. Therefore, large axial displacements decrease the 2P excitation intensity and degrade the axial resolution.

In wide-field TF, the laser power loss along the *y* direction is negligible because the beam shape on the LCOS-SLM is linear along the *x* direction. However, in TM-ML-TF, the illumination beam on the LCOS-SLM expands along the y direction, resulting in a loss of laser power in the *y* as well as the *x* direction. Thus, the reduction in 2P excitation intensity at large axial displacements by TM-ML-TF CGH is greater than that by TF-CGH.

The axial response of TM-ML-TF is given by:[4]

$$R_{TM}(z) = \frac{1}{\sqrt{1+\left(z/z_{Rx}\right)^2}\sqrt{1+\left(z/z_{Ry}\right)^2}} = \frac{1}{1+\left(z/z_{Rx}\right)^2},$$

where $z_{Rx} = z_{Ry}$ = FWHM / 2. If the effective NA of TM-ML-TF is the same as that of TF, the FWHM for TM-ML-TF is $\sqrt{3}$ times smaller than that for TF. Therefore, in TM-ML-TF CGH, the degradation of axial resolution due to the decrease in effective NA at large axial displacements is smaller than in TF CGH.

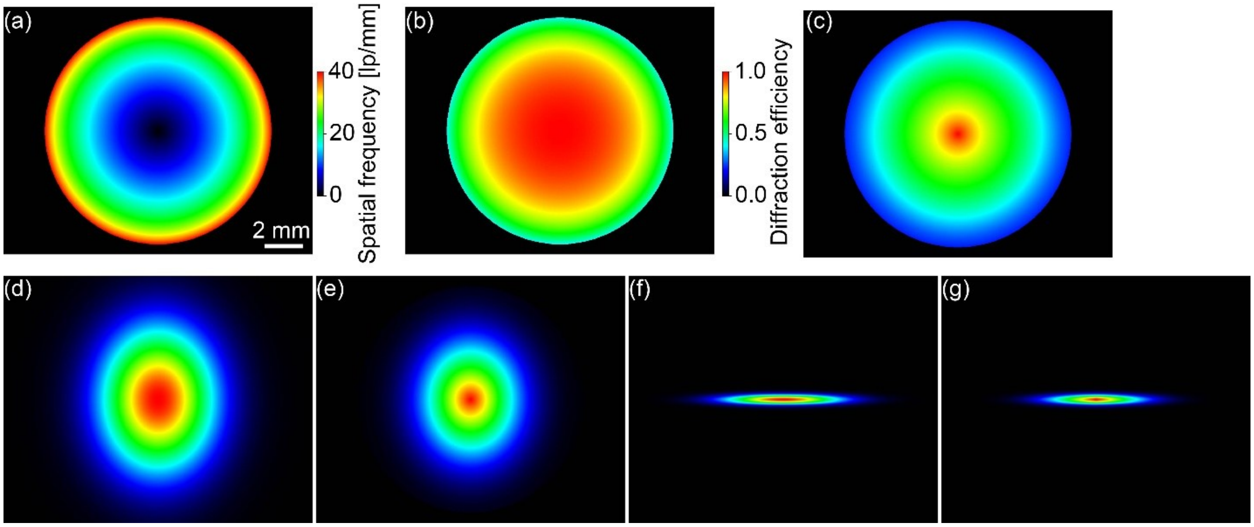

Figure S7. (a) Calculated local spatial frequency for 155-μm axial displacement. (b, c) Diffraction efficiencies for 155-μm axial displacement obtained by theory (b) and experiment (c). (d, e) Gaussian beam profiles with diameters of 8.27 mm (x) and 10.4 mm (y) without (d) and with (e) considering the diffraction efficiency for 155-μm axial displacement. (f, g) Gaussian beam profile with diameters of 8.27 mm (x) and 0.69 mm (y) without (f) and with (g) considering the diffraction efficiency for 155-μm axial displacement. Scale bar is 2mm.

References

1. G. Faini *et al*., "Ultrafast light targeting for high-throughput precise control of neuronal networks," Nat. Commun. **14**, 1888 (2023).
2. T. Ishikawa *et al*., "Fringe- and speckle-free holographic patterned illumination using time-multiplexed temporal focusing," Appl. Phys. Express **15**, 042005 (2022).
3. S. Yang *et al.,* "Three-dimensional holographic photostimulation of the dendritic arbor," *J. Neural Eng.* **8** 046002 (2011).
4. Q. Song *et al*., "Two-dimensional spatiotemporal focusing of femtosecond pulses and its applications in microscopy," Rev. Sci. Instrum. **86**, 083701 (2015).
